# Environmental enrichment as a multisystem modulator of neurotransmitter function: mechanisms, plasticity, and therapeutic implications

**DOI:** 10.3389/fnmol.2026.1927924

**Published:** 2026-10-16

**Authors:** Gaurav Singhal, Bernhard T. Baune

**Affiliations:** 1 Department of Otolaryngology - Head and Neck Surgery, University of Wisconsin, Madison, WI, United States; 2 Department of Psychiatry, University of Münster, Münster, Germany; 3 Department of Psychiatry, University of Melbourne, Melbourne, VIC, Australia

**Keywords:** brain, environmental enrichment, adrenergic, cholinergic, dopaminergic, glutamatergic, histaminergic, serotonergic

## Abstract

Environmental enrichment (EE) has emerged as a powerful non-pharmacological strategy for reshaping brain function through experience dependent plasticity. By integrating sensory, cognitive, motor, and social stimulation, EE provides a biologically relevant framework for understanding how external environments influence neurotransmitter systems that govern mood, cognition, stress adaptation, and neurodegeneration. In this review, we examined the effects of EE on major neurotransmitter pathways, including serotonergic, dopaminergic, glutamatergic, GABAergic, cholinergic, adrenergic, and histaminergic systems. We conducted a structured narrative search of PubMed, supplemented by Google Scholar reference list searching and informed by PRISMA reporting principles. The literature search covered studies published through 4 September 2026. The initial search, completed in February 2025, included 136 articles. A supplemental update screened 108 PubMed records and identified seven additional eligible primary studies, resulting in an updated evidence synthesis comprising 143 articles. Across predominantly preclinical studies, EE was consistently associated with enhanced neurogenesis, synaptic plasticity, stress resilience, and improved behavioral and cognitive outcomes, accompanied by neurotransmitter related changes across multiple brain systems. The most robust evidence supported modulation of serotonergic, dopaminergic, glutamatergic, and GABAergic pathways, whereas adrenergic and histaminergic effects were less well characterized and often indirect. Mechanistically, these effects converged on altered receptor and transporter expression, neurotrophic signaling, CREB dependent transcriptional regulation, and epigenetic remodeling. Collectively, the evidence positions EE as a multisystem regulator of neurotransmitter function with potential relevance to mood disorders, neuroinflammation, neurodegeneration, stroke, addiction, and cognitive decline. Nevertheless, the field remains limited by the predominance of animal data and by substantial heterogeneity in enrichment paradigms. Future translational and clinical studies are needed to define the most effective EE components, timing, and intensity for therapeutic application in humans.

## 1 Introduction

Environmental enrichment (EE), which includes exposure to increased sensory, cognitive, motor, and social stimulation, has emerged as a potent non-pharmacological approach to influence neurotransmitter system function and neuroplasticity (Singhal et al., 2014; Han et al., 2023). Understanding the mechanisms behind brain plasticity and resilience is critical in addressing mood disorders and neurodegenerative diseases, particularly those that impact cognition and emotional regulation. Neurotransmitter systems, including the aminergic, glutamatergic, GABAergic, and cholinergic systems, play fundamental roles in regulating brain function, mood, cognition, and neuroplasticity. Understanding how EE modulates these pathways may therefore inform therapeutic strategies aimed at enhancing brain resilience and plasticity in these conditions.

The major neuromodulatory neurotransmitter systems, including the serotonergic, dopaminergic, noradrenergic, and cholinergic systems, regulate mood, arousal, reward processing, attention, learning, and memory. Serotonin plays a central role in emotional regulation and stress responses (Hensler, 2010; Puglisi-Allegra and Andolina, 2015), and serotonergic mechanisms remain important therapeutic targets in mood disorders (Cipriani et al., 2018). Dopamine contributes to reward processing, attention, and executive function (Nieoullon, 2002; Jovanoski et al., 2023), with altered dopaminergic signaling implicated in several neuropsychiatric conditions (Chen et al., 2021). Norepinephrine and epinephrine regulate arousal and physiological responses to stress (Chu et al., 2024), particularly through the locus coeruleus–norepinephrine system (Ross and Van Bockstaele, 2021). The cholinergic system similarly supports attention, learning, memory, and hippocampal plasticity through nicotinic and muscarinic acetylcholine receptors (Ellis et al., 2006; Hasselmo, 2006; Maurer and Williams, 2017; Echeverria et al., 2023). Dysregulation of these neurotransmitter systems has been implicated in depression, anxiety, and other neuropsychiatric disorders, as well as Parkinson’s and Alzheimer’s diseases (Caspi et al., 2010; Newman-Tancredi et al., 2012; Kalia and Lang, 2015; Ferreira-Vieira et al., 2016; Kudryavtseva et al., 2017; Šimić et al., 2017; Cabana-Domínguez et al., 2022; Chen et al., 2022; McGrath et al., 2022; Pourhamzeh et al., 2022; Akyuz et al., 2025).

Glutamatergic and GABAergic systems provide the principal excitatory and inhibitory control of neuronal networks, respectively, and their coordinated activity is essential for maintaining excitatory-inhibitory balance. Glutamate supports learning, memory, long-term potentiation, and activity dependent synaptic remodeling (Meldrum, 2000; Traynelis et al., 2010). GABA is synthesized by glutamic acid decarboxylase, whose GAD65 and GAD67 isoforms contribute differently to synaptic and basal GABA production (Chattopadhyaya et al., 2007; Kajita and Mushiake, 2021; Bolneo et al., 2022). Through ionotropic GABA A and metabotropic GABA B receptors, GABA regulates neuronal excitability, network oscillations, and the timing of synaptic plasticity. Disruption of GABA synthesis or receptor signaling has been implicated in neurodevelopmental and psychiatric disorders, including autism spectrum disorder and schizophrenia (Charych et al., 2009; Fatemi et al., 2009; Yeung et al., 2018; Zhao et al., 2022), while GABAergic signaling also contributes fundamentally to neural development and neurological function (Bolneo et al., 2022). Together, these neuromodulatory and excitatory-inhibitory systems provide the neurochemical framework through which EE may influence cognition, emotional regulation, and brain plasticity.

This review discusses the potential of EE as a therapeutic avenue to modulate neurotransmitter expression in the brain, with profound implications for treating neurodegenerative disorders that affect mood. EE can influence the expression and epigenetic regulation of genes involved in serotonergic and dopaminergic signaling (Koh et al., 2007; Ryding et al., 2021; Arraes et al., 2024). EE can modulate epigenetic processes, such as DNA methylation and histone acetylation, leading to long-lasting changes in gene expression associated with neuroplasticity and cognitive function (de Sousa Fernandes et al., 2024). The inclusion of glutamate acknowledges its fundamental contributions to synaptic plasticity, a key mechanism underlying the cognitive and emotional benefits of EE, while serotonin and dopamine represent the most studied biogenic amines in this context. Although other aminergic systems, such as the histaminergic and adrenergic (epinephrine and norepinephrine) systems, also contribute to brain function, their specific modulation by EE has been less thoroughly explored in the literature. The central premise of this review is that EE should be understood as a systems-level regulator that engages multiple neurotransmitter pathways simultaneously rather than as a set of isolated single transmitter effects. We acknowledge these gaps in the literature and aim to provide a focused and critical evaluation of neurotransmitter systems that are most strongly linked to EE, while highlighting areas in need of further investigation. Throughout the review, evidence is described as direct when studies quantify neurotransmitter levels, receptor or transporter expression, or causal pathway manipulation, as convergent when multiple molecular and behavioral findings support neurotransmitter involvement, as indirect when inference is based on downstream markers or related pathways, and as hypothetical when a mechanistic link is biologically plausible but remains untested. Also, it is important to note that in the context of this narrative review, EE is used as an umbrella term for multi-component paradigms that may include voluntary physical activity, social interaction, novelty, sensory or olfactory stimulation, and cognitive complexity, although not every study incorporates all these components simultaneously.

## 2 Methodology

An important interpretive challenge is separating environmental complexity from voluntary exercise. Running wheel activity can independently influence hippocampal neurogenesis, BDNF signaling, and monoaminergic function, whereas many EE paradigms combine exercise with novelty and social stimulation. Accordingly, effects from uncontrolled multi-component paradigms are attributed here to the composite EE exposure, and attribution to a specific component is reserved for exercise-free or component-controlled designs (Rogers et al., 2016).

This article is a structured narrative review informed by selected PRISMA (Preferred Reporting Items for Systematic Reviews and Meta-Analyses) reporting principles. PRISMA was used to improve transparency in literature identification and selection; however, the review was designed for mechanistic and conceptual synthesis and was not prospectively registered or conducted as a formal systematic review or meta-analysis (Liberati et al., 2009; Moher et al., 2009). Consequently, the search counts show the evidence identification process rather than a claim of exhaustive systematic coverage.

### 2.1 Search and selection process

The initial PubMed search was run in February 2025 and covered records indexed from database inception through that date (Figure 1). The search was restricted to English language records, with no study design filter, using the following query: ((environmental enrichment OR enriched environment OR enriched environments) AND (brain OR neural OR neurology OR neuroplasticity) AND (neurotransmitter* OR serotonin OR dopamine OR norepinephrine OR noradrenaline OR epinephrine OR adrenaline OR glutamate OR GABA OR acetylcholine OR cholinergic OR histamine OR gene expression OR depression OR anxiety OR neurodegenerat*)). Google Scholar was searched at the same time using six query families: environmental enrichment neurotransmitter brain; environmental enrichment serotonin dopamine; environmental enrichment glutamate GABA; environmental enrichment acetylcholine norepinephrine histamine; environmental enrichment gene expression neuroplasticity; and environmental enrichment depression anxiety neurodegeneration. The first 100 relevance-ranked results for each query were inspected (600 result positions). Because Google Scholar rankings are dynamic and the same article can appear in more than one query, these 600 positions were not treated as 600 unique database records. Only potentially eligible citations were entered into EndNote 20 and combined with PubMed and citation search records. This produced an initial library of 291 candidate records; 19 duplicates were removed, 272 records underwent title/abstract screening, 83 were excluded at that stage, 189 full text articles were assessed, 53 were excluded, and 136 articles were included in the initial narrative synthesis. To update the review, the same PubMed query was rerun on 4 September 2026 for records published from 1 March 2025through 4 September 2026, yielding 108 records. The six Google Scholar query families and forward and backward citation searches were also repeated on 4 September 2026 to identify recent or newly indexed studies. Google Scholar results were used for supplementary identification and were not added to the PubMed yield. After title/abstract screening and eligibility assessment, seven eligible primary studies from the update were incorporated, producing an updated evidence synthesis of 143 articles.

**Figure 1:**
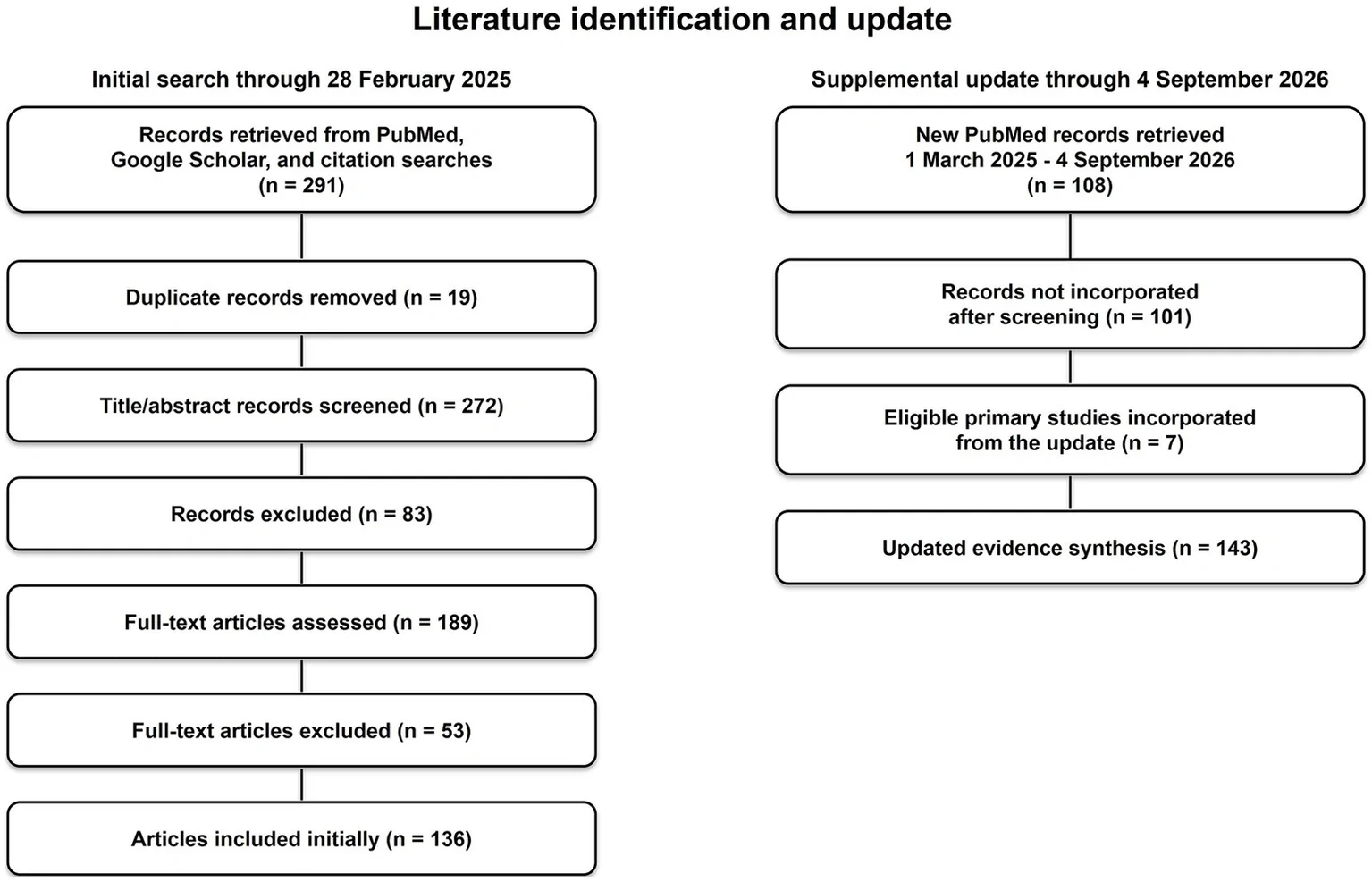
Literature identification and update for this structured narrative review. The initial search produced 291 candidate records, of which 19 duplicates were removed, 272 records were screened, 189 full texts were assessed, and 136 articles were included. The supplemental PubMed search retrieved 108 records published from 1 March 2025 through 4 September 2026, of which seven eligible primary studies were incorporated, producing an updated synthesis of 143 articles. For each Google Scholar update, the first 100 relevance-ranked results from each of six query families were inspected (600 result positions). However, since Google Scholar rankings are dynamic, these positions were not counted as 600 unique database records.

### 2.2 Inclusion and exclusion criteria

Primary studies were eligible for the evidence synthesis when they described an identifiable EE paradigm and directly assessed a neurotransmitter concentration, receptor, transporter, synthetic enzyme, innervation pattern, signaling pathway, or related functional measure. Animal and human studies were considered. Studies reporting behavioral outcomes alone without neurotransmitter related measurements, non-peer reviewed reports, studies lacking a sufficiently described enrichment exposure, and articles outside the neurotransmitter focused scope were excluded. Review articles were used only to provide background, identify additional primary studies, or support conceptual discussion and they were not treated as primary experimental evidence. Because this was a narrative rather than systematic review, formal risk-of-bias assessment and meta-analysis were not undertaken.

### 2.3 Interpretive handling of heterogeneous EE paradigms

For each finding, interpretation considered whether enrichment was exercise free, single component, or multi-component, the duration and developmental timing of exposure, brain region and disease context, and whether evidence was direct, convergent, indirect, or hypothetical. Where exercise and other EE components were combined without component controls, effects were attributed to the composite EE paradigm rather than to environmental complexity alone. This distinction is necessary because exercise can independently alter BDNF, neurogenesis, and monoaminergic signaling.

## 3 Neurotransmitter mediated neurogenesis and plasticity in response to EE

Adult neurogenesis, the process by which new neurons are generated in the adult brain, represents a key form of neuroplasticity influenced by environmental factors. Studies reveal that neurotransmitter systems, particularly serotonin, dopamine, and glutamate, play significant roles in mediating adult neurogenesis. For instance, serotonergic signaling, through pathways involving serotonin (5-HT) receptors, promotes cell proliferation and survival in the subventricular zone (SVZ) adjacent to the lateral ventricles (Vaidya et al., 2007). Similarly, dopaminergic inputs to the hippocampus influence the maturation and integration of newborn neurons (Takamura et al., 2014), while glutamatergic signaling supports synaptic connectivity and plasticity in these regions (Schlett, 2006).

EE induced plasticity can be conceptualized as a recurrent sequence: sensory, cognitive, social, and motor stimulation alters neuronal activity, interacting neurotransmitter systems gate intracellular signaling, BDNF-TrkB, CREB, and activity dependent transcription support neuronal survival and synaptic remodeling, and epigenetic mechanisms help stabilize some of these adaptations. Astrocytes and microglia further shape transmitter availability, excitatory-inhibitory balance, and neuroinflammation. This common framework provides the mechanistic bridge for the neurotransmitter specific evidence discussed in this review.

EE significantly enhances hippocampal neurogenesis in rodents, leading to improvements in learning, exploratory behavior, and locomotion (Kempermann et al., 2002). These behavioral benefits are tightly linked to neurotransmitter system engagement and downstream molecular signaling. The serotonergic system has been shown to mediate the anxiolytic and anti-depressive effects of EE by promoting neurogenesis, including in serotonin transporter knockout rats, although the rescue of depressive behaviors may be incomplete (Sbrini et al., 2020). Likewise, dopaminergic modulation through EE enhances dendritic spine density and synaptic strength, while simultaneously downregulating D2 receptor expression, a compensatory plasticity response that facilitates integration of new neurons (Gabriel et al., 2020). Glutamate-driven synaptic activity after EE promotes the survival of newly generated neurons and their incorporation into memory-associated circuits in the hippocampus (Segovia et al., 2006). These findings underscore the integrated nature of EE-induced neurogenesis, where neurotransmitter systems not only initiate neurogenic cascades but also support structural and functional maturation of new neurons.

## 4 EE as a modulator of neuromodulatory neurotransmitter systems

In the brain, neuromodulatory pathways, including dopaminergic, serotonergic, noradrenergic systems, histaminergic and cholinergic systems, play a central role in regulating neural circuits, synaptic plasticity, arousal, mood, learning, memory, and higher cognitive functions. Within this broader group, dopamine, serotonin, norepinephrine, epinephrine, and histamine are aminergic neurotransmitters, whereas acetylcholine constitutes a distinct neuromodulatory system. These pathways are dynamically influenced by environmental factors, which can modulate their activity and contribute to adaptive changes in brain function. EE may therefore promote neuroplasticity, emotional regulation, cognitive performance, and stress resilience through coordinated effects across multiple neuromodulatory systems.

EE has been demonstrated to enhance dopaminergic neurotransmission in both healthy and disease models, particularly in models of Parkinson’s disease, where it increases dopamine release and receptor sensitivity, offers neuroprotective effects and improves motor function (Alcalá-Zúniga et al., 2024). Similarly, EE modulates serotonergic pathways by increasing serotonin release, altering receptor expression, and enhancing downstream signaling, which correlates with improved mood regulation and reduced anxiety-like behavior (Rogers et al., 2017). EE may also influence noradrenergic signaling involved in arousal, stress adaptation, and memory. Evidence concerning the histaminergic system remains limited and is derived largely from metabolic and indirect epigenetic findings, whereas cholinergic studies indicate effects on acetylcholine signaling, cholinergic neuron preservation, learning, and memory. Together, these findings suggest that modulation of neuromodulatory neurotransmission is an important mechanism through which EE influences brain function.

Conditions such as schizophrenia (Peitl et al., 2020), bipolar disorder (Levinson and Young, 2022), and depression (Soga et al., 2021) have been linked to dysregulated aminergic neurotransmission in specific brain regions. Reduced dopaminergic activity in the prefrontal cortex is associated with cognitive deficits in schizophrenia, whereas impaired serotonergic signaling is linked to mood dysregulation in treatment resistant depression. Likewise, EE has also been reported to enhance noradrenergic neurotransmission in models of AD, potentially improving memory and reducing amyloid plaque accumulation. Histaminergic dysfunction may affect arousal, cognition, and mood, while cholinergic deficits are strongly associated with cognitive decline in neurodegenerative disorders. These observations support the potential of EE as a non-pharmacological strategy for modulating neuromodulatory pathways across neuropsychiatric and neurodegenerative conditions. For readability, the sections below consider the main evidence, proposed mechanisms, and key implications or limitations for each neuromodulatory system.

### 4.1 EE and the serotonergic system: evidence, mechanisms, and key implications

EE plays a critical role in modulating the serotonergic system, which is central to emotional regulation, cognitive functions, and neural development. Given its ability to influence serotonin signaling, EE has emerged as a promising non-pharmacological therapeutic intervention for cognitive and affective disorders.

On a behavioral level, EE has demonstrated anxiolytic and antidepressant effects across various genetic and disease models. In 5-HT1A receptor knockout mice, EE without exercise produced anxiolytic effects, underscoring its potential in reducing anxiety-related behaviors (Rogers et al., 2016). Similarly, in serotonin transporter (5-HTT) knockout mice, EE alleviated innate anxiety, although it did not fully rescue depressive-like behaviors, emphasizing the selective and partial effects of EE within the serotonergic system (Rogers et al., 2017). The impact of EE on depression has been demonstrated in a rat model of early-life seizures, where EE reversed a depression-like state and restored the expression of the serotonin receptor 5B (*Htr5B*) gene (Koh et al., 2007). In line with this, emerging evidence suggests that EE can attenuate depressive-like behaviors, a phenomenon closely linked to modulation of serotonin pathways (Chen et al., 2024; Vega-Rosas et al., 2024).

The influence of EE extends beyond behavior modifications to induce neurobiological changes. For example, in red junglefowl chicks, EE altered the expression of serotonergic genes such as tryptophan hydroxylase-1, an enzyme which is involved in serotonin synthesis, with corresponding changes in impulsivity (Ryding et al., 2021). Similarly, in rat models, postnatal serotonin depletion resulted in memory impairments and anxiety disorders, both of which were mitigated by EE, supporting its role in correcting serotonergic dysregulation during critical developmental periods (Saadati et al., 2023). Additionally, in animal models where prenatal antipsychotic exposure impaired hippocampal plasticity in adult offspring, EE in adulthood reversed structural deficits such as dendritic retraction and spine loss in the dentate gyrus, emphasizing that EE restores plasticity through serotonergic mechanisms (Wang et al., 2025).

The interplay between genetic predispositions and environmental factors is key in determining the efficacy of EE. Serotonin transporter knockout rats exposed to EE displayed improved neuroplasticity and reduced depression- and anxiety-like behaviors, illustrating how EE modulates serotonergic markers and plasticity in a gene-dependent manner (Sbrini et al., 2020).

At the cognitive level, EE enhances both cognitive and emotional performance by influencing neurotransmitter systems, including serotonin. In models of psychiatric disorders, EE improved outcomes via serotonergic pathways involved in cognitive and emotional processing (Rogers et al., 2019). In Huntington's disease models, EE reduced anxiety-related behaviors, although its effects on depression-like behaviors were limited and did not alter 5-HT1A receptor gene expression, suggesting a selective influence on emotional phenotypes (Renoir et al., 2013). Notably, combining EE with venlafaxine (an SNRI) potentiates antidepressant responses through parvalbumin interneurons and perineuronal net remodeling, implicating serotonergic and noradrenergic pathways (Coutens et al., 2023). This suggests a synergistic mechanism where EE may potentiate serotonin-mediated antidepressant responses.

However, the efficacy of EE is context-dependent and may vary with genetic background, developmental stage, and baseline serotonergic function. For example, the limited modulation of 5-HT1A receptors gene expression in Huntington’s disease models indicates that the effects of EE may not generalize across all serotonergic pathways (Renoir et al., 2013). Moreover, the duration of EE exposure appears critical, as 3-week EE has been associated with changes in serotonin levels in the frontal cortex, correlating with measurable behavioral outcomes, whereas short-term EE (24 h) can improve memory independently of serotonin changes (Leger et al., 2015).

Recent evidence further supports the impact of EE on serotonin related neuroplasticity. A key study by Karayol et al. (2021) demonstrated that serotonin receptor 4 (5-HT4R) in the hippocampus modulates mood and anxiety, with EE-like stimulation promoting 5-HT4R-dependent neuroplasticity and antidepressant-like effects. Extending these findings, Arraes et al. (2024) showed that 50 days of EE in a two-hit schizophrenia model produced hippocampal demethylation of the serotonin transporter gene (*Slc6a4*), enhancing neurogenesis and reducing microglial activation, thereby representing a novel epigenetic mechanism underlying EE's serotonergic effect. Furthermore, Bezerra da Silva et al. (2024) demonstrated in a rat study that post-weaning EE specifically increases 5-HT levels in the hippocampus and prefrontal cortex, reducing anxiety- and depression-like behaviors through long-term enhancement of serotonergic responsivity. A 2026 mouse study further linked EE after social isolation stress to normalization of hippocampal 5-HT, SERT, and 5-HT3A receptor measures and to SERT-TGFB1 dependent histone serotonylation associated with BDNF transcription (Sowndharya and Rajan, 2026).

### 4.2 EE and the dopaminergic system: evidence, mechanisms, and key implications

The dopaminergic system, known for its role in cognitive functions such as attention and executive control, has shown notable benefits from EE. Research has demonstrated that EE can protect dopaminergic neurons, particularly in models of Parkinson's disease. For example, exposure to EE in PD models has been associated with the upregulation of the DJ1 protein, a critical factor in dopamine regulation and neuroprotection, leading to improved behavioral outcomes (Alcalá-Zúniga et al., 2024). In addition, studies using the 1-methyl-4-phenyl-1,2,3,6-tetrahydropyridine (MPTP)-induced PD model reveal that EE increases the expression of tyrosine hydroxylase and neurotrophins while downregulating proNGF and the p75 neurotrophin receptor, indicating a multifaceted protective mechanism against dopaminergic neuron degeneration (Cho and Kang, 2020). Corroborating these findings, a recent study demonstrated that EE promotes recovery from neurotoxin-induced deficits in PD models by enhancing dopaminergic neuron survival and functionality, while simultaneously reducing neuroinflammation, a known contributor to neurodegenerative pathology (Alcalá-Zúniga et al., 2024).

EE also plays a role in modulating dopaminergic gene expression linked to behavioral outcomes. It has been shown to reduce FosB expression in the dopaminergic system, which is commonly associated with addiction-related behaviors (Zhang et al., 2014; Salinas-Velarde et al., 2021). This suggests that EE might help modulate reward-related pathways, potentially reducing addictive behaviors. Moreover, research on red junglefowl chicks found that EE influences the expression of dopamine receptor genes (*DRD1* and *DRD2*), correlating with impulsivity, further supporting EE's role in regulating behavioral traits through dopaminergic modulation (Ryding et al., 2021). In alignment with these behavioral insights, EE has been shown to alleviate anxiety-like behaviors in rodent models, with the dopaminergic system playing a pivotal mediating role, pointing to dopamine-dependent pathways in emotional regulation (Del Arco et al., 2007; Zhu and Grace, 2022).

On a molecular level, EE has demonstrated its ability to modulate key transcriptional processes involved in neuroprotection. EE activates immediate early genes such as *EGR1* and *NURR1*, which are implicated in protecting dopaminergic neurons (Wassouf et al., 2018). Furthermore, it has been observed that EE increases the expression of growth-associated protein-43 (GAP-43) in the substantia nigra, which suggests enhanced neurite outgrowth and axonal regeneration as mechanisms for reducing dopaminergic neuron damage (Yuan et al., 2018). Recent studies also reveal that EE can alter dopamine signaling within the mesolimbic circuit and the hippocampus by modulating the expression and phosphorylation of DARPP-32, a key mediator of dopamine signal transduction (Grimm et al., 2018; Wang et al., 2025). These molecular modifications bear significance for cognitive and motivational outcomes. Additionally, EE promotes internalization of striatal dopamine transporters, contributing to enhanced synaptic plasticity and neuronal communication (Kim et al., 2016; Thanos et al., 2016; Wassouf et al., 2018).

The broader implications of EE's effects on the dopaminergic system extend beyond disease models. Genetic factors play a role in moderating the outcomes of EE interventions. For example, the expression of the dopamine D2 gene can influence the impact of EE on both lifespan and behavior, highlighting the importance of genetic predispositions in determining EE efficacy (Thanos et al., 2016). Additionally, the therapeutic outcomes of EE appear to depend on the type of enrichment stimulus provided, physical, social, or cognitive, which may differentially influence dopaminergic pathways and their downstream behavioral effects (Gabriel et al., 2020). Duration of EE exposure also appears to be a crucial determinant of outcomes, with longer interventions producing more robust effects on behavior and synaptic plasticity in adult mice (Khalil, 2024). Moreover, the benefits of EE are not limited to behavioral and neurochemical modulation, it also promotes neurogenesis and dendritic remodeling through activity-dependent gene expression changes, contributing to structural plasticity in dopaminergic circuits (Alarcón et al., 2023). This variability emphasizes the necessity of customizing EE protocols based on the targeted dopaminergic outcomes and underlying genetic factors.

Recent advances further illuminate the scope of EE's dopaminergic effects. Zhu and Grace (2022) identified a critical prepubertal developmental window (postnatal days 21–40) during which EE prevents increased dopamine neuron population activity in a neurodevelopmental rat model of schizophrenia risk. Notably, EE applied in adulthood failed to confer the same protection (Zhu and Grace, 2022). This finding has significant implications for the timing of EE interventions in at-risk populations. Additionally, Vashisht et al. (2025) demonstrated that EE attenuates reinstatement of heroin seeking behavior and reverses heroin induced upregulation of mesolimbic ghrelin receptors in the ventral tegmental area and nucleus accumbens, broadening EE's relevance to addiction and reward pathway disorders.

Collectively, these findings highlight that EE-induced modulation of the dopaminergic system is multifaceted, involving behavioral, molecular, structural, and epigenetic processes. This broad impact supports the therapeutic potential of EE for neuropsychiatric and neurodegenerative disorders, especially those involving dopamine dysregulation.

### 4.3 EE and the adrenergic system: evidence, mechanisms, and key implications

The adrenergic system, driven by the catecholamines epinephrine and norepinephrine, is central to the regulation of stress response, attention, and executive function. Originating primarily in the locus coeruleus, norepinephrine is released widely throughout the brain, affecting regions such as the prefrontal cortex, amygdala, and hippocampus. This system operates through alpha- (α1, α2) and beta-adrenergic (β1, β2, β3) receptors, each mediating distinct physiological and cognitive processes. The adrenergic system's role in modulating arousal, memory, and emotional regulation underscores its relevance to EE interventions.

EE has been shown to significantly influence the adrenergic system, particularly through its effects on β-adrenergic signaling and noradrenaline release in the brain. This interaction is crucial for understanding how environmental factors can modulate genetic and neurobiological pathways associated with cognitive and behavioral outcomes. For example, EE enhances β-adrenergic signaling, which plays a protective role against microglial inflammation induced by amyloid-β oligomers. This is achieved through increased norepinephrine levels and β2-adrenergic receptor expression in the hippocampus, which are disrupted by amyloid-β in standard housing conditions (Xu et al., 2018). Mice in EE conditions showed similar microglial protection when administered β-adrenergic agonists, while this protection was lost with β-adrenergic antagonists, highlighting the critical role of β-adrenergic receptors in mediating the anti-inflammatory effects of EE (Xu et al., 2018).

EE also selectively increases the functional response of pre-synaptic NMDA receptors, which modulate noradrenaline release in the hippocampus. This upregulation is associated with improved learning and memory, suggesting that EE enhances cognitive functions through adrenergic pathways (Grilli et al., 2009). The efficacy of NMDA receptors is augmented in EE conditions, although the affinity remains unchanged, indicating a specific enhancement of receptor function rather than expression (Grilli et al., 2009).

Additional indirect evidence suggests that EE may influence adrenergic function through epigenetic modifications. For example, EE reduces DNMT activity, potentially also demethylating promoter regions of β-adrenergic receptors genes (e.g., *ADRB1*, *ADRB2*) (de Sousa Fernandes et al., 2024; Torres et al., 2025). EE elevates histone H3 acetylation (H3K14ac) in brain regions like the prefrontal cortex, promoting chromatin relaxation and β-AR gene activation. This modification is linked to enhanced CREB phosphorylation, a transcription factor critical for adrenergic signaling (Baroncelli et al., 2010; Torres et al., 2025).

Research has confirmed that epigenetic mechanisms such as DNA methylation and histone acetylation play a central role in β_2_-adrenergic receptor (β_2_AR) gene expression regulation in T-helper cells, indicating that adrenergic modulation via environmental input extends beyond the central nervous system to the immune interface (McAlees et al., 2011). Another study has linked environmental stressors to epigenetic alterations that influence gene expression and physiological reactivity, reinforcing the dynamic sensitivity of the adrenergic system to external stimuli (Kubota, 2016).

EE has also been shown to enhance BDNF transcription in the hippocampus through both transcriptional and epigenetic mechanisms (Costa et al., 2023), further linking the adrenergic system to neurotrophic regulation of synaptic function. Additionally, research on environmental stressors shows that adverse stimuli can produce epigenetic changes that impair adrenergic signaling, reinforcing the idea that EE and stress represent opposing ends of an environmental continuum in shaping gene expression profiles (Kubota, 2016). Emerging perspectives on lifestyle interventions suggest that even behavioral practices like meditation, considered forms of environmental enrichment, can modulate gene expression via epigenetic changes relevant to adrenergic function and cognitive control (Venditti et al., 2020).

Supporting these findings, Terrier et al. (2024) demonstrated that long-term olfactory enrichment increases noradrenergic innervation in brain regions associated with olfactory processing and memory in older mice, with associated improvements in spatial memory and cognitive flexibility (Terrier et al., 2024). This study directly quantified noradrenergic structural plasticity as an EE outcome, implicating noradrenaline as a molecular link between sensory enrichment and cognitive reserve. Additionally, Xu et al. (2022) showed that EE following early life stress attenuates PTSD-like behavioral abnormalities through epigenetic regulation of the hippocampus and amygdala, regions heavily innervated by the locus coeruleus noradrenergic system, via altered histone acetylation patterns (Xu et al., 2022).

Taken together, these findings highlight the adrenergic system as a key mediator of EE's cognitive and anti-inflammatory benefits. At present, however, the evidential base is mixed in strength. Some findings are direct, including beta-adrenergic signaling, noradrenaline release related mechanisms, and noradrenergic structural plasticity (Grilli et al., 2009; Xu et al., 2018; Terrier et al., 2024), whereas several proposed epigenetic links remain indirect or inferential. Emerging evidence also suggests that some EE induced responses may be sex dependent. EE increased hippocampal and pituitary BDNF levels in male mice but increased adrenal tyrosine hydroxylase and phenylethanolamine N-methyltransferase levels in females, indicating distinct neurotrophic and peripheral catecholaminergic responses between the sexes (Herselman et al., 2026). Further studies are needed to confirm these sex specific effects, determine whether they persist after enrichment ends, and establish their relevance to conditions involving adrenergic dysregulation, including PTSD and other stress related disorders.

### 4.4 EE and the histaminergic system: evidence, mechanisms, and key implications

The histaminergic system, mediated by histamine released from hypothalamic neurons, plays a critical role in arousal, cognition, and the sleep-wake cycle. The influence of EE on genes that regulate the expression of histamine, and its receptors remain to be fully elucidated. However, indirect evidence suggests EE may influence histaminergic circuits through behavioral and epigenetic pathways, though direct evidence remains limited (Mohsen et al., 2014).

EE and histamine receptors, particularly H1 receptors, independently influence behavior and cognition. For example, EE is known to enhance cognitive and motor functions, counteracting the effects of harmful stimuli such as social isolation and neurotoxicity. It has been shown to improve memory formation by stimulating synaptic plasticity, similar to the effects of histone acetylation, which is crucial for learning and memory (Peixoto and Abel, 2013; Penney and Tsai, 2014). Likewise, histamine, through its receptors (H1, H2, and H3), regulates various physiological functions, including arousal, cognition, locomotor activity, and exploratory behavior (Mickley, 1986; Tashiro et al., 2002; Mohsen et al., 2014), which are also modulated by EE. The absence of H1 receptors in mice has been linked to impaired locomotor activity and reduced exploratory behavior, indicating the role of histamine in regulating these behaviors (Inoue et al., 1996). Research has also shown that environmental factors, such as stress and light/dark cycles, can modulate the circadian regulation of plasma histamine levels and these changes are mediated by the mast cell clock and can influence allergic reactions and other histamine-related functions (Nakamura et al., 2017). Although these overlaps suggest potential interaction, direct experimental evidence of EE modulating histaminergic receptor expression or activity remains to be demonstrated.

EE reduces DNA methyltransferase (DNMT) activity in the hippocampus, a key enzyme responsible for gene silencing via CpG methylation (de Sousa Fernandes et al., 2024). Although direct studies on histaminergic genes (e.g., *HDC*, H1R–H4R) are limited, it is plausible that reduced DNMT activity could demethylate promoter regions of histamine-synthesizing enzymes or receptors, enhancing their transcription. Similarly, EE upregulates glucocorticoid receptor expression via histone acetylation in the amygdala, a region with dense histaminergic innervation (de Sousa Fernandes et al., 2024). This suggests possible crosstalk between EE-induced histone remodeling and histaminergic signaling in stress and arousal pathways.

Notably, a recent metabolomic profiling study by Zhang et al. (2024) provided the first direct evidence that EE elevates brain histamine levels: aged mice exposed to EE showed increased hippocampal histamine and tyrosine metabolites alongside reduced glutamate concentrations, coinciding with reversal of depressive-like symptoms, reduced neuroinflammation, and enhanced neurogenesis (Zhang et al., 2024). Although histamine was not the primary focus of this study, its unbiased metabolomic design identified histaminergic changes as part of EE's broad neurochemical normalization profile in aged brains. These findings open a new research avenue investigating whether EE directly modulates tuberomammillary nucleus activity and histamine receptor (H1–H4) expression, particularly in the context of geriatric depression and age-related cognitive decline.

Hence, while direct evidence linking EE to histaminergic gene expression is sparse, its epigenetic effects on DNMTs and histone modifiers provide a plausible framework for such interactions. At present, however, this evidence should be regarded as preliminary, with the strongest direct support coming from metabolomic rather than receptor or gene-expression studies. Further research is needed to directly characterize EE's effects on histamine synthesis enzymes (HDC), histamine receptors (H1R-H4R), and tuberomammillary nucleus neuronal activity. This represents one of the most significant gaps in the current EE literature and warrants dedicated mechanistic investigation, particularly given the histaminergic system's role in arousal, wakefulness, and mood regulation.

### 4.5 EE and the cholinergic system: evidence, mechanisms, and key implications

The cholinergic system, driven by the neurotransmitter acetylcholine (ACh), is essential for numerous cognitive and physiological processes, including learning, memory, attention, and motor coordination. This system operates through two types of receptors: nicotinic acetylcholine receptors (nAChRs) and muscarinic acetylcholine receptors (mAChRs). Nicotinic receptors are ionotropic, mediating rapid responses, while muscarinic receptors are metabotropic, involved in slower, modulatory processes. The basal forebrain, specifically the nucleus basalis of Meynert and medial septum, is the primary source of cholinergic projections to the cortex and hippocampus, regions critical for cognitive function.

EE has been shown to significantly influence cholinergic gene expression. The cholinergic system, particularly the expression of cholinergic receptors and enzymes, is modulated by environmental factors, and EE provides a complex and stimulating environment that can enhance cognitive abilities and emotional resilience. For example, EE has been found to enhance the expression of α7-nAChR in the hippocampus, which is associated with improved cognitive function in post-stroke cognitive impairment (PSCI) models. This receptor plays a role in reducing inflammation and promoting neurological recovery, suggesting that EE can activate the cholinergic anti-inflammatory pathway, thereby improving cognitive outcomes in PSCI rats (Mei et al., 2020). Similarly, in the little Indian field mouse, EE has been shown to influence the splicing of acetylcholinesterase (AChE) by upregulating micro-RNA-183, which shifts the splicing from the synaptic AChE-S form to the read-through AChE-R form. This shift is associated with reduced anxiety-like behavior, indicating that EE can modulate cholinergic transmission and stress responses through alterations in AChE expression (Ragu Varman et al., 2013).

Lifelong EE has been shown to protect the basal forebrain cholinergic system, which is crucial for spatial memory and learning. This protection may contribute to the delayed onset of learning deficits and the prevention of age-related memory retrieval alterations, highlighting the role of EE in maintaining cholinergic function across the lifespan (Harati et al., 2013). EE also elevates BDNF and IGF-1 levels, which promote cholinergic neuron survival and synaptic integration. For example, BDNF-TrkB signaling enhances ChAT expression and acetylcholine release in the hippocampus, improving spatial memory in rodent models (Paban et al., 2011).

In a landmark multi-omic study, Nam et al. (2024) directly quantified neurotransmitter changes in 5XFAD Alzheimer's disease model mice following 4 weeks of EE, demonstrating significant increases in acetylcholine concentrations in both the frontal cortex and hippocampus, alongside elevated serotonin levels (Nam et al., 2024). Given that cholinergic deficits are a hallmark of Alzheimer's pathology, these findings suggest that EE may exert cholinergic supportive effects with therapeutic relevance to Alzheimer's disease, although direct evidence for disease modification remains limited. Supporting this therapeutic perspective, Mohd Sahini et al. showed in a comprehensive review that EE consistently improves cognitive function in rodent dementia models through reduced neuroinflammation, enhanced neuronal plasticity, and prevention of amyloid-beta accumulation at early disease stages, mechanisms that directly interact with and preserve the cholinergic system (Sahini et al., 2024).

While EE consistently enhances multiple aspects of cholinergic function, the specific molecular mechanisms linking environmental stimulation to cholinergic receptor upregulation and acetylcholine synthesis remain incompletely understood. Sex differences, age at EE onset, and the duration of enrichment exposure are likely important moderating variables. Therefore, further research is needed to optimize EE protocols and to elucidate the full range of mechanisms through which EE promotes cholinergic gene expression and cognitive health, particularly with a view toward clinical translation in Alzheimer's disease and other cholinergic dementias.

## 5 EE as a modulator of principal excitatory and inhibitory neurotransmitter systems

Glutamatergic and GABAergic neurotransmission provides the principal excitatory and inhibitory control of neuronal activity, respectively. Their coordinated interaction maintains the excitatory-inhibitory balance required for stable neural-network function, synaptic plasticity, learning, memory, and adaptive behavior. Glutamate promotes neuronal excitation and activity dependent plasticity through receptors such as NMDA and AMPA receptors, whereas GABA constrains excessive excitation and regulates the timing and synchronization of neuronal activity through GABA A and GABA B receptors (Meldrum, 2000; Traynelis et al., 2010; Bolneo et al., 2022).

EE can influence both components of this balance by modifying receptor expression, synaptic organization, neurotrophic signaling, and neuronal-network activity. Its effects are not necessarily unidirectional. Depending on the brain region, developmental stage, disease model, and enrichment protocol, EE may enhance excitatory plasticity, strengthen inhibitory control, or restore a disrupted balance between the two systems. This context dependent regulation may contribute to the beneficial effects of EE on cognition, emotional regulation, stress resilience, and recovery from neurological injury.

The following sections examine the evidence that EE modulates glutamatergic and GABAergic neurotransmission, the mechanisms underlying these effects, and their principal functional and therapeutic implications.

### 5.1 EE and the glutamatergic system: evidence, mechanisms, and key implications

As the principal excitatory neurotransmitter system in the brain, the glutamatergic system plays a crucial role in cognitive function and synaptic plasticity. EE modulates glutamatergic signaling through changes in receptor expression, synaptic organization, and activity dependent plasticity. NMDA and AMPA receptors, which are central to glutamatergic neurotransmission, undergo EE induced changes in the expression of their constituent subunits (Lee et al., 2003; Bredy et al., 2004; Grilli et al., 2009). Enriched housing conditions have been shown to enhance the gene expression of the hippocampal AMPA GluR1 receptor subunit in immune challenged rats, suggesting that EE mitigates the adverse effects of repeated immune challenges through glutamatergic gene modulation (Mlynarik et al., 2004). Furthermore, EE improves hippocampal long-term potentiation (LTP), a process critical for learning and memory, through the modulation of mGluR5 signaling (Hullinger et al., 2015; Buschler and Manahan-Vaughan, 2017), a metabotropic glutamate receptor involved in synaptic efficacy and plasticity.

EE induces metaplastic changes in glutamatergic synapses across various brain regions, including the medial prefrontal cortex, nucleus accumbens, and hippocampus. These changes involve alterations in N-methyl-D-aspartate (NMDA) and α-amino-3-hydroxy-5-methyl-4-isoxazolepropionic acid (AMPA) receptor subunits and their associated scaffolding proteins, which influence synaptic receptor retention, hence the key mechanisms underlying the regulation of reward and memory processes (Pintori et al., 2024). In animal models where the NMDA receptor 1 is specifically knocked out in the hippocampus, EE has been shown to rescue memory deficits and alter the expression of genes involved in synaptic transmission and structure, demonstrating its capacity to reverse gene expression changes associated with memory impairments (Li et al., 2007).

This modulatory effect of EE appears to be region- and condition-specific. For example, in amblyopic rats, EE increases glucose metabolism and the expression of glucose transporter proteins in the visual cortex, potentially improving visual nerve conduction via enhanced glutamatergic activity (Xie et al., 2024). Likewise, in Sprague-Dawley rats, EE selectively enhanced excitatory glutamatergic synaptic transmission in layer II/III of the auditory cortex and induced auditory potentials and synaptic currents, improving sensory integration (Nichols et al., 2007). Region-specific effects of EE extend to the hippocampus, where EE modulates the expression of glutamate transporters such as Excitatory Amino Acid Carrier 1 (EAAC1) and increases NMDA receptor mRNA levels. These changes fine-tune glutamatergic signaling and contribute to cognitive functions like memory and learning (Andin et al., 2007).

Glial regulation adds another level of context. Astrocytic transporters determine extracellular transmitter availability: EAAT2/GLT-1 and EAAC1 constrain glutamate accumulation, whereas GAT-1 contributes to GABA clearance and the balance between tonic and phasic inhibition. EE related changes in these transport systems may therefore influence synaptic plasticity and recovery without requiring a uniform change in neuronal transmitter synthesis. Direct EE evidence is stronger for selected transporters and disease models than for a generalized astrocytic mechanism, so broader claims remain provisional (Andin et al., 2007; Lin et al., 2021; Pajarillo et al., 2021; Cuellar-Santoyo et al., 2023).

On a broader level, EE influences glutamatergic signaling through epigenetic mechanisms. For instance, changes in histone methylation and the expression of NMDA receptor subunits and glucocorticoid receptors have been observed, potentially enhancing sociability and stress resilience in enriched environments (Jeyaraj et al., 2021). EE has also shown neuroprotective effects in ischemic models, where preconditioning reduces infarct volume and short-term memory deficits by modulating the expression of glutamatergic receptors and inflammatory mediators (Goncalves et al., 2018). Together, these findings support the concept that EE can induce both protective and performance-enhancing changes in glutamatergic circuits across multiple brain areas.

A recent study by Martinez-Torres et al. (2024) further clarifies the glutamatergic basis of EE-mediated neurorehabilitation in stroke. Using a rat model of ischemic stroke, they demonstrated that post-stroke EE selectively increased hippocampal AMPA receptor GluA1 subunit levels and reduced infarct size, while NMDA receptor 1 expression was not significantly altered (Martínez-Torres et al., 2024). This dissociation suggests that AMPA receptor mediated synaptic plasticity, rather than NMDA receptor changes, is the primary glutamatergic substrate responsible for EE induced functional recovery following cerebrovascular injury. Furthermore, EE has been proposed as a neuroprotective strategy in Alzheimer's disease models through restoration of glutamatergic excitatory/inhibitory balance, upregulation of BDNF, SYN, and PSD95, and enhancement of long-term potentiation (Neves et al., 2024). Recent studies provide more direct mechanistic support. In stressed mice, EE preserved prefrontal GluA2 and NR1 expression, dendritic spine density, and neuronal firing, and receptor antagonism blocked the behavioral benefit (Xu et al., 2025). In ischemic rats, EE increased astrocytic GLT-1 by suppressing SMURF1 dependent ubiquitination, thereby enhancing glutamate uptake and reducing excitotoxic injury (Zhang et al., 2025).

While these findings provide compelling evidence for EE-mediated glutamatergic plasticity, further studies are necessary to delineate the precise intracellular mechanisms, duration-sensitive effects, and gene-environment interactions that mediate these changes. In particular, identifying individual variability and replicability across different experimental contexts will be critical to translating these insights into targeted therapeutic applications.

### 5.2 EE and the GABAergic system: evidence, mechanisms, and key implications

EE has been shown to significantly influence GABAergic gene expression, impacting various neurological and psychological conditions. The effects of EE on GABAergic systems are multifaceted, involving changes in neurotransmission, synaptic plasticity, and resilience to stress. These changes are crucial for understanding how environmental factors can modulate brain function and potentially serve as therapeutic interventions for neurological disorders. For example, EE during early postnatal development enhances GABAergic neurotransmission, promoting synapse maturation. This is evidenced by increased amplitude of miniature GABAergic postsynaptic currents and elevated levels of GABA(A) receptors in EE-reared mice, indicating enhanced synaptic transmission and maturation (He et al., 2010). In adulthood, EE can reverse pathological GABAergic inhibition. For instance, in a mouse model of Down syndrome, EE reduced excessive GABAergic inhibition, which was found to be associated with improved cognitive abilities, synaptic plasticity, and visual functions (Begenisic et al., 2011). This suggests that EE can modulate inhibitory neurotransmission to restore cognitive functions.

EE associated changes in parvalbumin (PV) must be interpreted by circuit rather than by direction alone. Reduced PV expression in the developing somatosensory cortex may reflect local circuit reorganization and altered critical period plasticity (Inzunza et al., 2003), whereas preservation or restoration of PV positive interneurons in the dorsal hippocampus or anterior cingulate cortex can support stress resilience, gamma oscillations, and relief of pain related anxiety (Begega et al., 2023; Ren et al., 2025). Similarly, reducing excessive tonic GABAergic inhibition after stroke is functionally distinct from strengthening phasic inhibition. These examples preclude describing all increases or decreases as generic “restoration”.

In the context of stress, chronic stress reduces parvalbumin positive GABAergic neurons in the dorsal hippocampus, while EE maintains and protects these populations, contributing to pro-resilient outcomes against stress induced depression and anxiety (Begega et al., 2023). EE has been proposed as a potential therapeutic strategy for stroke recovery by modulating GABAergic signaling. Specifically, EE reduces tonic GABA inhibition and enhances phasic inhibition, promoting network excitability and recovery. This effect is mediated by the GABA transporter GAT-1, highlighting a potential target for therapeutic intervention (Lin et al., 2021). In models of prenatal stress, EE restores impaired spatial memory and synaptic plasticity, with GABAergic modulation playing a crucial role. The use of GABAergic antagonists further enhances these effects, indicating that targeting GABAergic pathways can potentiate the benefits of EE (Aghighi Bidgoli et al., 2020).

Recent studies have further expanded the understanding of EE's GABAergic effects in novel clinical contexts. Ren et al. (2025) demonstrated that EE restored parvalbumin (PV)-positive GABAergic interneuron density in the anterior cingulate cortex in a neuropathic pain model, reversing SNI-induced depletion of PV puncta and restoring fast gamma oscillations (80–140 Hz) (Ren et al., 2025). EE improved anxiety-like behavior without affecting mechanical allodynia, supporting a specific role for PV-interneuron mediated cortical inhibitory control in pain associated anxiety while not establishing clinical efficacy. In an epilepsy model, Li et al. showed that EE enhanced adult born dentate granule cells were necessary and sufficient for reduced seizure susceptibility and identified an entorhinal cortex-dentate gyrus pathway engaging local hilar GABAergic interneurons. Selective activation of these neurons mimicked the EE associated protection, whereas their inhibition abolished it (Li et al., 2024). More recent circuit level evidence from an early life stress rat model showed that EE alleviated visceral pain and depression-like behaviors while normalizing CB1R associated activity in a prelimbic glutamatergic-anteroventral BNST GABAergic-PVN pathway, further illustrating context dependent coordination among excitatory, inhibitory, and neuromodulatory signaling (Huang et al., 2026).

While the positive effects of EE on GABAergic neurotransmission are well supported, the precise nature of these effects is highly context dependent. For instance, the interaction between genetic predispositions, such as those seen in Down syndrome or epilepsy models, and environmental factors can lead to differential outcomes, ranging from reduced inhibition to enhanced inhibitory tone (Inzunza et al., 2003; He et al., 2010; Begenisic et al., 2011; Aghighi Bidgoli et al., 2020; Lin et al., 2021; Begega et al., 2023). The specific timing, duration, and components of EE exposure are critical variables that influence the extent of GABAergic modulation. Understanding these sources of variability is essential for optimizing EE protocols for specific neurological disorders and translating preclinical findings to clinical practice.

## 6 Discussion

This review synthesizes predominantly preclinical evidence, together with limited human evidence, indicating that EE can modulate multiple neurotransmitter systems in the brain. Figure 2 summarizes the organizing model of this review. EE components first alter activity across interacting neuromodulatory and excitatory-inhibitory networks. These systems converge on neurotrophic signaling, CREB dependent transcription, neurogenesis, synaptic remodeling, glial regulation, and epigenetic processes. Brain region, cell type, age, sex, genotype, disease state, exposure timing, and EE composition determine whether a measured change is adaptive, compensatory, or ineffective. Supporting this context dependent multisystem framework, Dong et al. demonstrated that enriched environment treatment altered the coordinated expression of 81 genes associated with dopaminergic, adrenergic, serotonergic, glutamatergic, GABAergic, and cholinergic systems, with the magnitude and persistence of these effects varying according to age, brain region, and BDNF status (Dong et al., 2020). The framework therefore emphasizes coordinated and context dependent network regulation rather than a uniform increase or decrease in individual transmitters.

**Figure 2:**
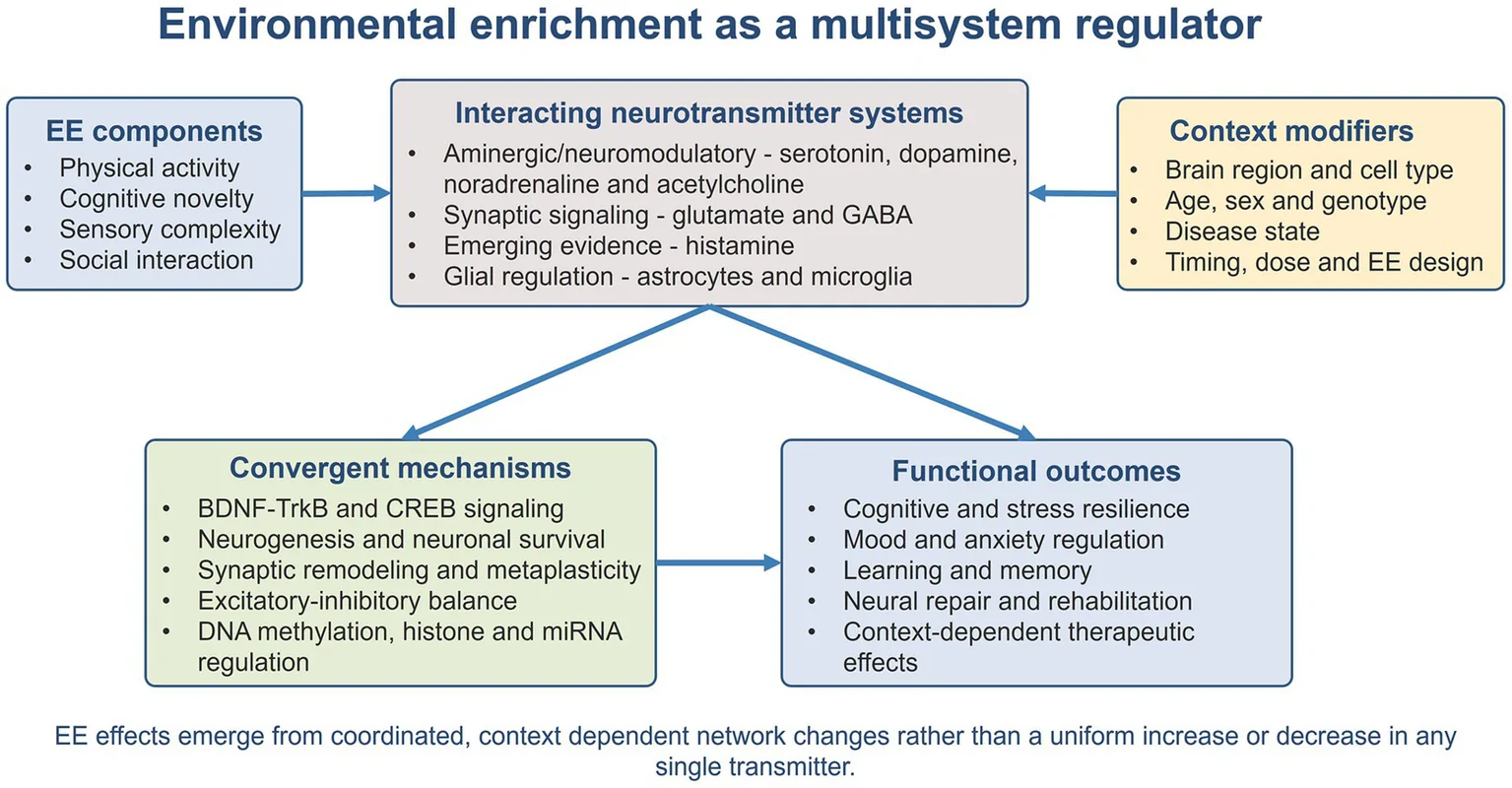
Integrative framework for environmental enrichment as a multisystem regulator. Environmental enrichment components engage interacting neurotransmitter and glial systems that converge on common plasticity mechanisms and functional outcomes. Context modifiers determine the direction and significance of observed changes. Arrows represent conceptual integration rather than proof of a single linear causal pathway.

Cumulative evidence supports EE as a promising experimental framework for studying neuroplasticity, emotional regulation, and resilience in preclinical models, with possible relevance to stroke recovery, neuropathic pain, and addiction that requires clinical validation. Overall, the strongest and most consistent evidence supports EE mediated modulation of serotonergic, dopaminergic, glutamatergic, GABAergic, and cholinergic systems, whereas adrenergic findings are best regarded as emerging and partly indirect, and histaminergic findings remain preliminary with only limited direct support (Table 1).

**Table 1:** Strength of evidence for the impact of EE on neurotransmitter systems.

Neurotransmitter system	EE effects summary	Strength of evidence	Key references
Serotonergic	Modulates gene expression (e.g., *HTR5B*), increases serotonin release, influences mood and anxiety, partially rescues deficits in SERT KO models.	Strong (multiple behavioral, molecular, gene expression studies)	Sbrini et al. (2020); Rogers et al. (2017); Rogers et al. (2016); Koh et al. (2007); Ryding et al. (2021); Saadati et al. (2023); Wang et al. (2025); Renoir et al. (2013)
Dopaminergic	Enhances dopamine release, receptor sensitivity, and neuroprotection in Parkinson’s disease models, modulates behavior and gene expression (e.g., *DRD1*, *DRD2*).	Strong (preclinical, molecular, behavioral, epigenetic studies)	Del Arco et al. (2007); Takamura et al. (2014); Gabriel et al. (2020); Cho and Kang (2020); Ryding et al. (2021); Alcalá-Zúniga et al. (2024); Salinas-Velarde et al. (2021); Yuan et al. (2018); Thanos et al. (2016)
Glutamatergic	Alters receptor expression (NMDA, AMPA), enhances synaptic plasticity, supports memory and sensory processing.	Strong (behavioral, molecular, region-specific studies)	Segovia et al. (2006); Lee et al. (2003); Mlynarik et al. (2004); Buschler and Manahan-Vaughan (2017); Pintori et al. (2024); Nichols et al. (2007); Andin et al. (2007); Jeyaraj et al. (2021); Darna et al. (2015)
GABAergic	Enhances synaptic inhibition and GABA receptor expression, rebalances excitatory-inhibitory tone, promotes resilience to stress.	Moderate to strong (context- and genotype-dependent effects)	Sbrini et al. (2020); Segovia et al. (2006); He et al. (2010); Begenisic et al. (2011); Begega et al. (2023); Aghighi Bidgoli et al. (2020)
Cholinergic	Increases ACh levels, ChAT expression, and α7-nAChR activity, protects cholinergic neurons, supports learning and memory.	Moderate (behavioral, molecular, context- and age-dependent)	Mei et al. (2020); Ragu Varman et al. (2013); Harati et al. (2013); Paban et al. (2011); Hilario et al. (2016)
Adrenergic	Enhances β-adrenergic receptor signaling, norepinephrine release, and neuroimmune modulation. Effects potentially mediated in partvia epigenetic changes.	Preliminary (some molecular and epigenetic data, fewer direct studies)	Xu et al. (2018); Grilli et al. (2009); McAlees et al. (2011)
Histaminergic	Limited direct evidence, overlaps in behavioral domains, potential epigenetic modulation of *HDC* and receptor (H1R–H4R) genes.	Limited (speculative and indirect links)	Tashiro et al. (2002); Peixoto and Abel (2013); Penney and Tsai (2014); Mohsen et al. (2014); Inoue et al. (1996); Haas and Panula (2003)

Evidence grading reflects the breadth and directness of the available literature. Strong indicates multiple primary studies with direct neurotransmitter, receptor, transporter, or causal pathway evidence supported by convergent functional or behavioral findings across models. Moderate indicates several supportive studies, but with narrower replication, fewer direct molecular measures, or greater context dependence. Limited or preliminary indicates sparse, emerging, or predominantly indirect evidence. HTR5B, 5-Hydroxytryptamine receptor 5B; SERT KO, serotonin transporter knockout; DRD1, dopamine receptor D1; DRD2, dopamine receptor D2; NMDA, N-Methyl-D-Aspartate (receptor); AMPA, α-Amino-3-Hydroxy-5-Methyl-4-Isoxazolepropionic Acid (receptor); GABA, gamma-aminobutyric acid; ACh, acetylcholine; ChAT, choline Acetyltransferase; nAChR, nicotinic acetylcholine receptor; HDC, histidine decarboxylase; H1R-H4R, histamine receptor H1-H4.

A system level summary of the molecular targets, brain regions, experimental models, and behavioral outcomes associated with EE across neurotransmitter systems is provided in Table 2. This overview highlights that EE does not produce a uniform neurotransmitter response but rather engages distinct molecular pathways depending on the neurotransmitter system, brain region, developmental stage, disease model, and enrichment paradigm.

**Table 2:** Summary of EE effects on major neurotransmitter systems in the brain.

Neurotransmitter system	Key molecules/genes affected	Brain regions	Animal models	Behavioral outcomes	References
Serotonergic	TPH1, SERT (*Slc6a4*), 5-HT1A, 5-HTT, Htr5B, 5-HT4R; ↑5-HT levels in hippocampus/PFC	Hippocampus, PFC, amygdala, raphe nuclei	SERT-KO mice/rats, 5-HT1A-KO mice, seizure models, HD transgenic mice, schizophrenia two-hit model	↓ anxiety-like behavior, antidepressant effects, improved cognition, restored serotonergic tone after early life stress	Koh et al. (2007); Rogers et al. (2016); Rogers et al. (2017); Sbrini et al. (2020); Karayol et al. (2021); Ryding et al. (2021); Arraes et al. (2024); da Silva et al. (2024)
Dopaminergic	TH, DAT, *DRD1*, *DRD2*, DJ1, NURR1, EGR1, GAP-43, proNGF, ΔFosB; ↑DA release and receptor sensitivity	Striatum, substantia nigra, PFC, nucleus accumbens (NAc), VTA, hippocampus	MPTP-PD model, MAM schizophrenia model, 6-OHDA model, addiction/heroin model, red junglefowl	Improved motor function; dopaminergic neuroprotection; reduced addictive/drug-seeking behavior; modulated impulsivity	Del Arco et al. (2007); Takamura et al. (2014); Darna et al. (2015); Thanos et al. (2016); Wassouf et al. (2018); Cho and Kang (2020); Gabriel et al. (2020); Salinas-Velarde et al. (2021); Zhu and Grace (2022); Jovanoski et al. (2023)
Noradrenergic/adrenergic	β-AR (*ADRB1*, *ADRB2*), NE release, pre-synaptic NMDA-R; ↑β2-AR expression; ↑CREB phosphorylation	Hippocampus, PFC, amygdala, locus coeruleus, olfactory regions	Amyloid-β oligomer models, PTSD/early-life stress models, aged mice	Improved learning/memory; reduced neuroinflammation; anti-amyloid microglial protection; reduced PTSD-like behaviors	Grilli et al. (2009); McAlees et al. (2011); Xu et al. (2018); Ross and Van Bockstaele (2021); Xu et al. (2022)
Glutamatergic	GluA1 (AMPA-R), NMDA-R1, NR2A, mGluR5, EAAC1, PSD95, SGK; ↑LTP, ↑AMPA-R subunits	Hippocampus, PFC, NAc, visual cortex, auditory cortex, spinal cord	Immune-challenged rats, NMDA-R1-KO (CA1-specific), ischemic stroke models, amblyopic rats, AD models	Enhanced LTP; improved spatial memory and learning; neuroprotection after ischemia; restored visual function	Meldrum (2000); Lee et al. (2003); Schlett (2006); Segovia et al. (2006); Andin et al. (2007); Nichols et al. (2007); Traynelis et al. (2010); Darna et al. (2015); Gerhard et al. (2016); Buschler and Manahan-Vaughan (2017); Yuan et al. (2018); Jeyaraj et al. (2021); McGrath et al. (2022); Pintori et al. (2024); Ren et al. (2025)
GABAergic	GAD65/67, *GABRA1*, *GABRB2*, PV interneurons, GAT-1, GABA-A receptor subunits; ↓excessive inhibition or ↑inhibitory tone (context-dependent)	Hippocampus, somatosensory cortex, anterior cingulate cortex (ACC), dentate gyrus, dorsal hippocampus	Down syndrome model (Ts65Dn), prenatal stress model, stroke model, epilepsy model, neuropathic pain (SNI) model	Reduced anxiety; improved cognition and synaptic plasticity; anti-seizure effects; stress resilience; pain-comorbid anxiety relief	Inzunza et al. (2003); Segovia et al. (2006); Charych et al. (2009); Fatemi et al. (2009); He et al. (2010); Begenisic et al. (2011); Yeung et al. (2018); Aghighi Bidgoli et al. (2020); Lin et al. (2021); Zhao et al. (2022); Begega et al. (2023)
Cholinergic	α7-nAChR, AChE (AChE-S/R splicing), ChAT, VAChT, mAChR; ↑ACh levels; ↑BDNF-TrkB signaling	Hippocampus, basal forebrain, cortex, nucleus basalis of Meynert	Post-stroke cognitive impairment (PSCI) rats, Mus booduga field mouse, aged rodents, 5XFAD AD model	Improved cognitive function; reduced anxiety; neuroprotection of cholinergic neurons; delayed age-related memory decline	Hasselmo (2006); Paban et al. (2011); Harati et al. (2013); Ragu Varman et al. (2013); Ferreira-Vieira et al. (2016); Hilario et al. (2016); Maurer and Williams (2017); Mei et al. (2020)
Histaminergic	Histamine metabolism (↑histamine levels via metabolomics); H1–H4R (indirect); *HDC*; crosstalk with DNMT/histone pathways	Hippocampus, hypothalamus (tuberomammillary nucleus), amygdala	Aged mice (depression model); indirect evidence from epigenetic EE studies	Mood improvement; arousal modulation; indirect evidence through EE epigenetic crosstalk; research gap identified	Mickley (1986); Inoue et al. (1996); Tashiro et al.(2002); Peixoto and Abel (2013); Mohsen et al. (2014); Penney and Tsai (2014); Nakamura et al. (2017); Vargas-Romero et al. (2019); Nam et al. (2024)

PFC, prefrontal cortex; VTA, ventral tegmental area; NAc, nucleus accumbens; TH, tyrosine hydroxylase; SERT, serotonin transporter; TPH1, tryptophan hydroxylase-1; ChAT, choline acetyltransferase; GAD, glutamic acid decarboxylase; PV, parvalbumin; LTP, long-term potentiation; BDNF, brain-derived neurotrophic factor; EE, environmental enrichment; NT, neurotransmitter; KO, knockout; AD, alzheimer's disease; PD, Parkinson's disease; HD, huntington's disease; PSCI, post-stroke cognitive impairment; SNI, spared nerve injury; HDC, histidine decarboxylase. Up arrows (↑) indicate increase, and down arrows (↓) indicate decrease. Each row synthesizes evidence from multiple publications rather than representing a single experimental study. Since the sex of the experimental animals varies among the cited studies and because some cited sources are reviews or do not report sex, a single sex designation cannot be assigned accurately at the row level. Sex dependent findings and related limitations are discussed in the main text.

EE consistently upregulates serotonergic function in regions critical for emotional regulation, including the hippocampus and prefrontal cortex. Beyond simple increases in 5-HT levels, EE modulates serotonin transporter expression through epigenetic mechanisms, including demethylation of the *Slc6a4* promoter, and enhances serotonin receptor expression and synaptic turnover (Rogers et al., 2017; Sbrini et al., 2020; Arraes et al., 2024; da Silva et al., 2024). These mechanisms likely underline the anxiolytic and antidepressive behavioral outcomes observed across diverse preclinical models. The gene-environment interaction is particularly salient. EE mediated serotonergic benefits vary according to baseline genetic architecture, as evidenced by differential responses in serotonin transporter knockout models (Renoir et al., 2013; Sbrini et al., 2020).

EE exerts neuroprotective effects on dopaminergic neurons, most prominently in Parkinson's disease models, through upregulation of tyrosine hydroxylase, neurotrophins, and transcription factors such as EGR1 and NURR1 (Wassouf et al., 2018; Cho and Kang, 2020; Alcalá-Zúniga et al., 2024). A critical developmental window has been identified, i.e., prepubertal EE prevents mesolimbic dopamine dysregulation in neurodevelopmental schizophrenia risk models, whereas adult-onset EE does not confer equivalent protection (Zhu and Grace, 2022). EE also attenuates addiction related dopaminergic adaptations by reducing Delta FosB expression and reversing mesolimbic ghrelin receptor upregulation following drug exposure (Salinas-Velarde et al., 2021; Vashisht et al., 2025), broadening EE's relevance to reward pathway disorders.

EE induces metaplastic changes at glutamatergic synapses across multiple brain regions, including upregulation of AMPA and NMDA receptor subunits, mGluR5-mediated LTP enhancement, and improved glutamate transporter expression (Lee et al., 2003; Bredy et al., 2004; Mlynarik et al., 2004; Andin et al., 2007; Li et al., 2007; Nichols et al., 2007; Grilli et al., 2009; Hullinger et al., 2015; Buschler and Manahan-Vaughan, 2017; Goncalves et al., 2018; Jeyaraj et al., 2021; Pintori et al., 2024; Xie et al., 2024). In stroke models, EE selectively increases hippocampal GluA1 (AMPA) subunit levels while NMDA receptor expression remains largely unchanged (Martínez-Torres et al., 2024), suggesting that AMPA receptor mediated plasticity is the primary glutamatergic substrate for EE-induced neurorehabilitation after cerebrovascular injury. Epigenetic modulation of NMDA receptor subunits and glucocorticoid receptors further extends these glutamatergic effects to sociability and stress resilience (Jeyaraj et al., 2021).

EE modulates GABAergic inhibition in a region- and context-specific manner. It promotes GABAergic synapse maturation and reduces excessive inhibition in conditions such as Down syndrome (He et al., 2010; Begenisic et al., 2011), while decreasing GABAergic tone in the dorsal hippocampus to promote stress resilience (Begega et al., 2023). Critically, EE restores PV-positive interneuron density in the anterior cingulate cortex, mediating anxiolytic effects through restoration of gamma oscillations (Ren et al., 2025), and EE induced adult neurogenesis contributes to anti-seizure protection through GABAergic interneuron recruitment (Gu, 2025). These findings position GABAergic circuit modulation as a central mechanism linking environmental stimulation to both cognitive and emotional outcomes.

EE consistently enhances cholinergic function through upregulation of alpha7-nAChR (Mei et al., 2020), preservation of ChAT expression (Harati et al., 2013), and BDNF-TrkB-mediated support of cholinergic neuron survival (Paban et al., 2011). Direct measurement of neurotransmitter concentrations in AD model mice following EE revealed significant increases in acetylcholine in frontal cortex and hippocampus (Nam et al., 2024), suggesting that EE may exert cholinergic supportive effects analogous to some goals of cholinergic therapies. Given that cholinergic deficit is a hallmark of Alzheimer's pathology, these findings strengthen the case for EE as a promising adjunctive or supportive intervention, although direct disease-modifying evidence remains limited.

The adrenergic literature provides emerging but not yet fully mature evidence for EE mediated modulation. EE enhances beta-adrenergic signaling to reduce amyloid-beta induced microglial neuroinflammation (Xu et al., 2018), upregulates presynaptic NMDA receptor function modulating hippocampal noradrenaline release (Grilli et al., 2009), and engages epigenetic mechanisms including DNMT reduction and histone acetylation to regulate adrenergic receptor gene expression (Baroncelli et al., 2010; Torres et al., 2025). Olfactory EE specifically increases noradrenergic innervation in aged mice, associated with improved spatial memory and cognitive flexibility (Terrier et al., 2024), while EE following early life stress normalizes hippocampal and amygdalar epigenetic landscapes disrupted by hyperactivation of locus coeruleus noradrenergic circuits (Xu et al., 2022). However, the epigenetic component of this model remains more indirect and mechanistically inferential than the receptor and innervation findings and should therefore be interpreted cautiously until tested more directly.

The histaminergic system remains the least studied of the neurotransmitter systems in the context of EE, representing a significant gap in the field. Although no direct evidence yet exists for EE regulation of histaminergic receptor or gene expression, EE-associated reductions in DNMT activity (de Sousa Fernandes et al., 2024; Torres et al., 2025) may plausibly extend to histaminergic genes—a hypothesis that warrants direct investigation. Current support is therefore best viewed as preliminary rather than established. Crucially, metabolomic profiling of EE treated aged mice revealed elevated brain histamine and tyrosine metabolites coinciding with reversal of geriatric depressive symptoms (Zhang et al., 2024), providing the first direct evidence of EE induced histamine metabolism changes. Future mechanistic studies targeting histamine synthesis (HDC), receptor expression (H1R-H4R), and tuberomammillary nucleus activity in response to EE are warranted.

A critical insight from this review is that EE does not act on neurotransmitter systems in isolation. Serotonin modulates glutamatergic and GABAergic neurotransmission (Ciranna, 2006), dopaminergic and cholinergic systems interact in basal ganglia circuits (Hilario et al., 2016), and adrenergic signaling regulates glutamate release through presynaptic NMDA receptors (Grilli et al., 2009). These cross-system interactions suggest that EE's broad spectrum neuroplastic effects arise from simultaneous, synergistic modulation of multiple neurotransmitter pathways, producing coherent changes in behavior, cognition, and mood that exceed what any single system intervention could achieve. Glial cells, including astrocytes (glutamate/GABA recycling) and microglia (neuroinflammation), also contribute to these multi-system effects (Singhal and Baune, 2017; Pajarillo et al., 2021; Kilb and Kirischuk, 2022; Cuellar-Santoyo et al., 2023), and their roles in mediating EE responses merit further investigation.

The therapeutic potential of EE spans diverse conditions, such as mood disorders, neurodegeneration, stroke, neuropathic pain, and addiction. Figure 3 and Table 3 summarize disease specific evidence linking EE mediated neurotransmitter modulation to functional outcomes across these models. This disease centered synthesis underscores that EE may be especially valuable in disorders characterized by convergent neurotransmitter dysregulation rather than isolated deficits in a single pathway.

**Table 3:** Disease models and EE mediated neurotransmitter modulation.

Disease/condition	NT system(s)	EE components used	Key molecular change	Functional outcome	References
Parkinson's disease	Dopaminergic cholinergic	Physical exercise, social interaction, novel objects	↑TH, ↑BDNF, ↑NURR1, ↑EGR1, ↑GAP-43, ↓DAT mRNA, ↓proNGF; ↑DJ1 protein; preserved ChAT, ↓AChE	Improved motor function; dopaminergic neuroprotection; partial cholinergic preservation	Hilario et al. (2016); Wassouf et al. (2018); Cho and Kang (2020); Alarcón et al. (2023)
Alzheimer's disease	Cholinergic glutamatergic serotonergic	Multi-component EE (exercise, novelty, social)	↑ACh (frontal cortex, hippocampus), ↑serotonin, ↑GluA1 (AMPA-R), ↑BDNF, ↑PSD95, ↓Aβ accumulation	Improved spatial memory; reduced Aβ plaques; enhanced synaptic plasticity; delayed cognitive decline	Ferreira-Vieira et al. (2016); Balthazar et al. (2018); Xu et al. (2018); Yuan et al. (2018); Neves et al. (2024)
Depression/anxiety	Serotonergic GABAergic Neurogenesis	Social enrichment, novelty, physical activity	↑5-HT in hippocampus/PFC; *Slc6a4*CpG demethylation; ↑BDNF; ↑GAD65/67; ↑DCX+ neurogenesis; ↑ARG1+ microglia	Anxiolytic and antidepressant behavioral effects; enhanced neuroplasticity; reversed depressive phenotype in aged mice	Kempermann et al. (2002); Rogers et al. (2017); Coutens et al. (2023); Chen et al. (2024); Khalil (2024); Zhang et al. (2024); Wang et al. (2025)
Schizophrenia	Dopaminergic Serotonergic	Prepubertal EE (critical window: PND 21–40); multi-component	↓DA neuron population activity in mesolimbic circuit; hippocampal *Slc6a4*demethylation; ↓microglial activation	Reduced sensorimotor gating deficits; normalized dopaminergic tone; improved social behavior	Zhu and Grace (2022); Arraes et al. (2024)
Stroke/cerebral ischemia	Glutamatergic GABAergic	Post-stroke multi-component EE	↑GluA1 (AMPA-R, hippocampus); ↓infarct volume; ↓tonic GABA inhibition; ↑GAT-1 expression; ↑phasic GABA inhibition	Improved motor and cognitive recovery; reduced infarct size; enhanced network excitability and rehabilitation	Goncalves et al. (2018); Mei et al. (2020); Han et al. (2023); Martínez-Torres et al. (2024)
Huntington's disease	Serotonergic	Multi-component EE (pre-symptomatic onset most effective)	Modulation of serotonergic receptors and circuits; selective emotional phenotype effects	Reduced anxiety-like behavior; limited effect on depression-like phenotype; timing-dependent efficacy	Renoir et al. (2013); Zajac et al. (2018)
Down syndrome	GABAergic	Early postnatal EE	↓excessive GABAergic inhibition; normalization of GABA-A receptor activity	Improved cognitive abilities; enhanced synaptic plasticity and visual cortex function	Begenisic et al. (2011)
Neuropathic pain (pain-comorbid anxiety)	GABAergic Glutamatergic	Multi-component EE	↑PV+ interneuron density in ACC; ↑gamma oscillations (80–140 Hz); ↓excessive glutamate release in spinal cord; ↑spinal GABAergic transmission	Reduced anxiety (without analgesic effect on allodynia); partial E/I balance restoration; pain-affect circuit dissociation	Ren et al. (2025)
Addiction/substance use disorder	Dopaminergic	Social + physical EE	↓ΔFosB in dopaminergic circuits; ↓mesolimbic ghrelin receptor (GHS-R1a) in VTA and NAc	Attenuated reinstatement of drug-seeking (heroin); modulated reward-related dopaminergic pathways	Zhang et al. (2014); Vashisht et al. (2025)
Epilepsy/seizures	GABAergic Neurogenesis	Multi-component EE	↑adult-born dentate granule cells (abDGCs); efferent projections from abDGCs activate hilar GABAergic interneurons	Reduced seizure frequency and severity; anti-epileptic effect mediated specifically by EE-induced neurogenesis	Gu (2025)
PTSD/early-life stress	Noradrenergic GABAergic	EE intervention post-stress exposure	↑H3K14ac in hippocampus; ↓H3K9ac/H4K12ac in amygdala; normalization of HAT/HDAC balance in LC-innervated regions	Reduced PTSD-like behavioral abnormalities; normalized stress-circuit histone landscape	Xu et al. (2022)
Geriatric depression	Histaminergic Serotonergic Neurogenesis	Multi-component EE in aged mice	↑hippocampal histamine and tyrosine metabolites (metabolomics); ↓glutamate; ↑DCX+ neurogenesis; ↑ARG1+ anti-inflammatory microglia; ↑BDNF	Reversed anhedonia; improved neurogenesis; reduced neuroinflammation; first direct EE-histamine metabolic link	Zhang et al. (2024)

PND, postnatal day; ACC, anterior cingulate cortex; VTA, ventral tegmental area; NAc, nucleus accumbens; abDGC, adult born dentate granule cell; LC, locus coeruleus; HAT, histone acetyltransferase; HDAC, histone deacetylase; E/I = excitatory/inhibitory; Aβ, amyloid-beta; DCX, doublecortin; EE: environmental enrichment. All data derived from preclinical (rodent) studies unless otherwise indicated. Each row synthesizes evidence from multiple publications rather than representing a single experimental study. Since the sex of the experimental animals varies among the cited studies and because some cited sources are reviews or do not report sex, a single sex designation cannot be assigned accurately at the row level. Sex dependent findings and related limitations are discussed in the main text.

**Figure 3:**
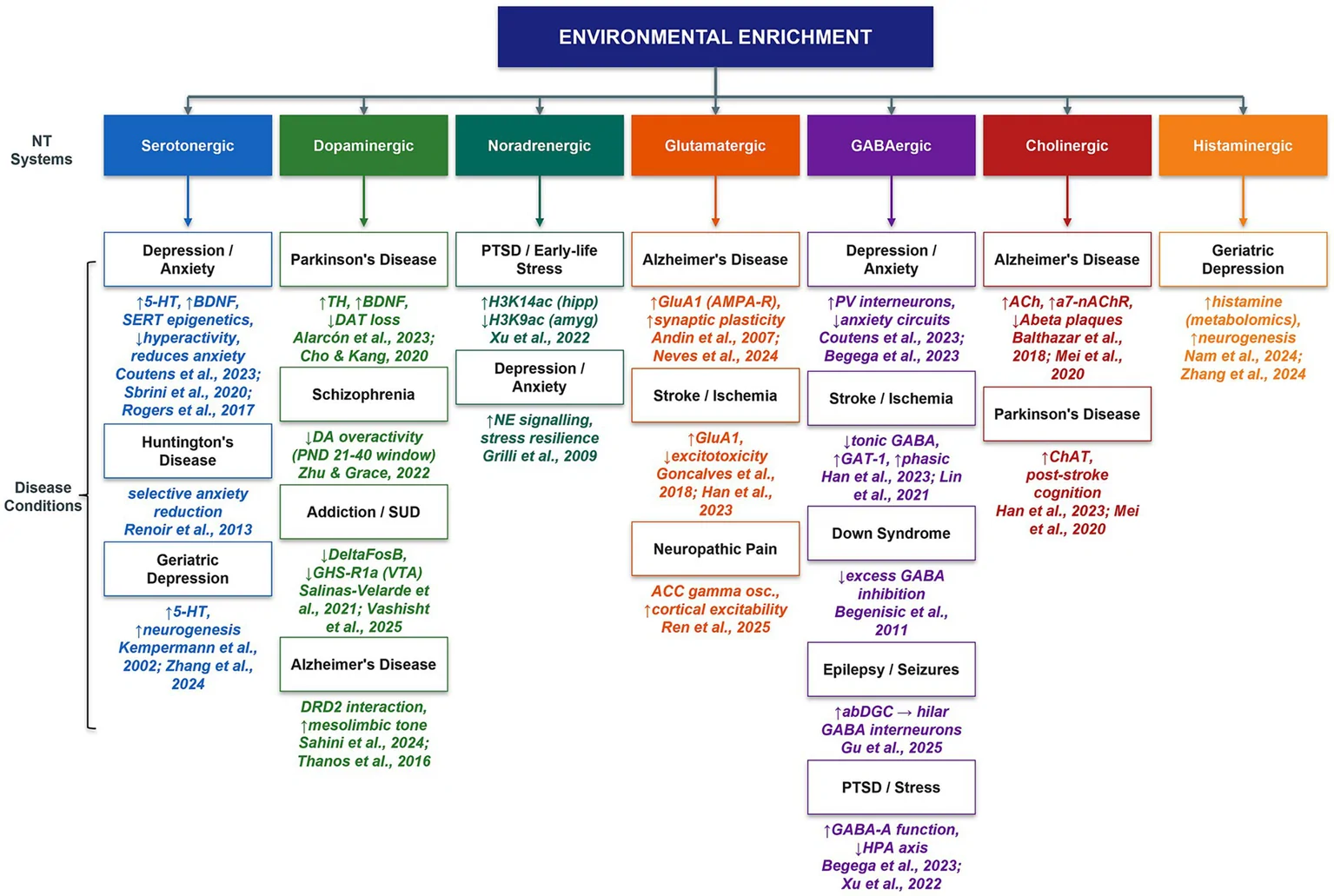
EE as therapeutic strategy: disease conditions and NT system targets. Disease conditions are listed under the NT systems that EE primarily modulates in that condition. abDGC, adult born dentate granule cell; SUD, substance use disorder; ACC, anterior cingulate cortex; VTA, ventral tegmental area.

One of the most compelling mechanistic themes emerging from recent work is the epigenetic programming of neurotransmitter systems by EE (Figure 4). Across neurotransmitter pathways, EE has been associated with changes in DNA methylation, histone acetylation, chromatin accessibility, and post-transcriptional regulation. These mechanisms may explain how relatively brief or developmentally timed environmental interventions produce durable changes in neurotransmitter gene expression and behavioral outcomes.

**Figure 4:**
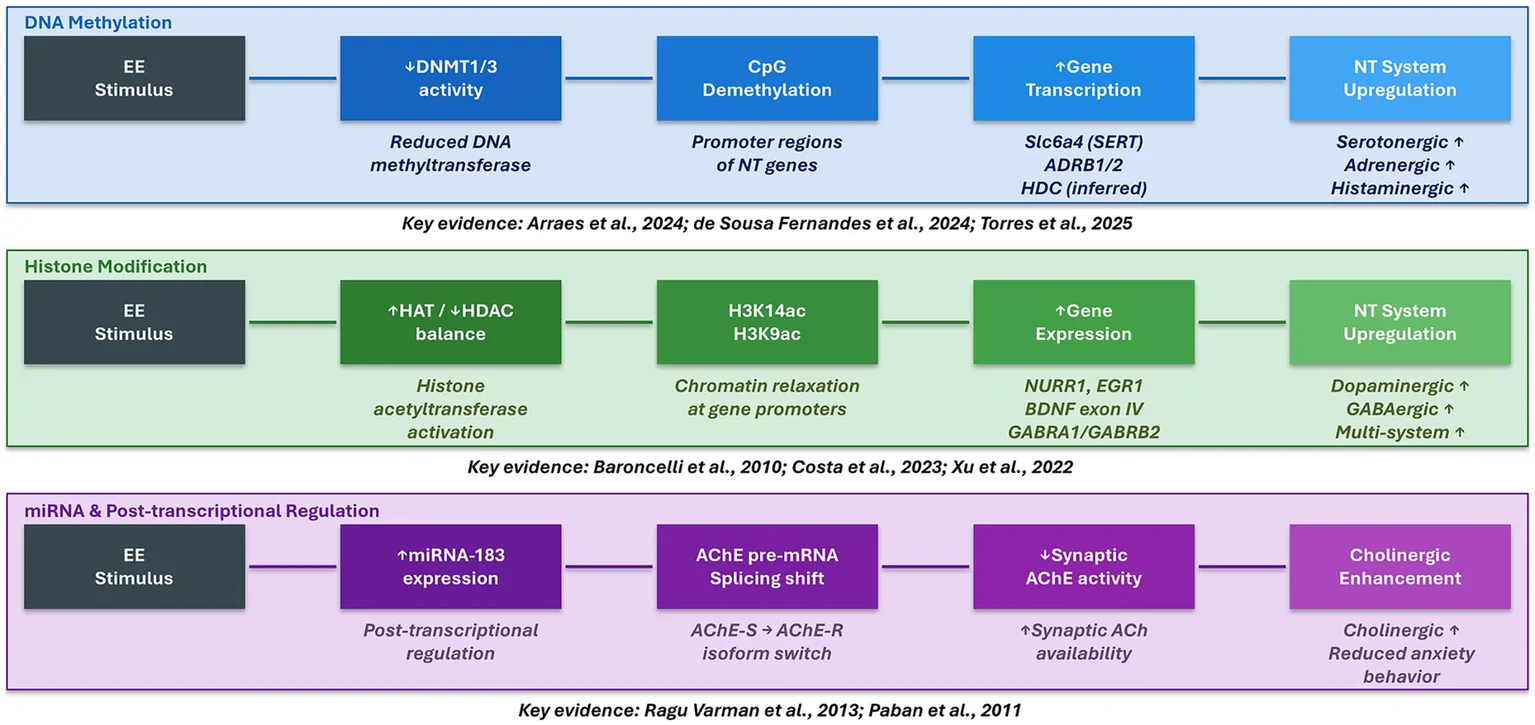
Epigenetic mechanisms underlying EE mediated neurotransmitter modulation. Cross-track interactions illustrate convergence across neurotransmitter systems. *Slc6a4* related methylation findings represent empirical EE evidence. *ADRB1/2* regulation is supported indirectly by EE associated epigenetic pathways and should not be interpreted as direct promoter level proof in the reviewed studies. *HDC* is a theoretical candidate only and direct EE induced regulation of the *HDC* promoter has not been demonstrated. BDNF upregulation (Track 2) potentiates serotonergic, dopaminergic, and cholinergic systems simultaneously. HAT, histone acetyltransferase; HDAC, histone deacetylase; DNMT, DNA methyltransferase; H3K14ac, histone H3 lysine-14 acetylation; AChE, acetylcholinesterase.

Direct human evidence remains limited and does not yet establish that the mechanisms observed in animal EE paradigms translate to clinical benefit. In a small human study, higher cognitive plus social enrichment scores correlated with serum BDNF, although the sample was limited and the design was observational (Vega-Rosas et al., 2024). A 2025 cross-sectional study of 50 adults with major depressive disorder likewise found that greater enrichment tended to accompany lower symptom severity, but the cognitive domain comparison was not statistically significant (Guiza-Zayas et al., 2025). Other cited sources map animal EE components onto human lifestyle factors or synthesize preclinical evidence rather than provide direct intervention evidence (Colavitta et al., 2023; Khalil, 2024; Sahini et al., 2024). Structured exercise, cognitive stimulation, social engagement, rehabilitation environments, sensory rich or natural settings, and digital platforms should therefore be regarded as candidate human analogs that require prospective clinical evaluation.

## 7 Limitations

Despite the substantial evidence reviewed here, several limitations warrant consideration. The majority of studies are preclinical and optimal enrichment parameters, such as duration, intensity, and components (physical, cognitive, social), for clinical translation remain to be defined. EE's effects are heterogeneous and context dependent, influenced by genetic background, age at exposure, sex, and disease model. Translation to human settings is further complicated by inter-individual variability in baseline activity, social context, comorbidity burden, adherence, and access to enriched environments. The histaminergic system remains poorly characterized in EE contexts, and sex-specific effects on neurotransmitter modulation are understudied. Future research priorities should include: (1) direct characterization of EE's effects on histaminergic gene expression and tuberomammillary nucleus activity, (2) sex stratified analyses of EE outcomes across neurotransmitter systems, (3) mechanistic investigation of the prepubertal critical window for dopaminergic EE effects, (4) multi-omic studies simultaneously profiling multiple neurotransmitter systems in response to EE, and (5) randomized controlled trials of EE inspired lifestyle interventions for mood disorders and neurodegeneration. Sex is a particularly important source of uncertainty because baseline neurotransmitter biology and responses to enrichment can differ between males and females, while many preclinical EE studies use one sex or do not report sex stratified outcomes. Sex dimorphic responses reported in disease models such as Huntington's disease (Zajac et al., 2018) caution against assuming equivalent effects across sexes. Study sex should therefore be extracted and displayed consistently in future evidence updates and where it is not reported or outcomes are not stratified, this absence should be explicit.

## 8 Conclusion

EE has emerged as a powerful non-pharmacological approach to modulating neurotransmitter systems and enhancing neuroplasticity. By influencing serotonergic, dopaminergic, adrenergic, glutamatergic, GABAergic, cholinergic, and histaminergic pathways, EE is associated with cognitive resilience, emotional regulation, and neural repair in predominantly preclinical studies. Among the neurotransmitter systems examined, the effects of EE on dopaminergic, serotonergic, glutamatergic, and GABAergic pathways are supported by stronger and more consistent experimental evidence, whereas findings related to adrenergic and histaminergic systems remain limited, preliminary, or indirect. EE has been shown to upregulate neurotrophic factors, alter epigenetic markers, and modulate the expression of neurotransmitter receptors, which supports further investigation of its relevance to mood disorders, neuroinflammation, neurodegeneration, and cognitive decline without establishing therapeutic efficacy in humans. These effects appear to be mediated by converging pathways involving BDNF signaling, CREB phosphorylation, and epigenetic remodeling, which operate across multiple neurotransmitter systems. While substantial evidence supports the benefits of EE on neurotransmitter systems, the effects remain context-dependent, influenced by genetic and environmental factors. Individual variability in response to EE, shaped by age, sex, genotype, baseline neurobiology, and type of enrichment, must be carefully considered when designing interventions. In translational terms, the most plausible human analogs of EE include combined programs of physical activity, cognitive stimulation, social engagement, rehabilitation, and enriched sensory or naturalistic environments, particularly for depression, dementia, Parkinson's disease, stroke recovery, and addiction related disorders. Future research should focus on optimizing EE protocols, understanding individual variability, and elucidating the precise molecular mechanisms underlying its impact on neurotransmitter regulation.

## References

[ref1] Aghighi Bidgoli F. Salami M. Talaei S. A. (2020). Environmental enrichment restores impaired spatial memory and synaptic plasticity in prenatally stress exposed rats: the role of GABAergic neurotransmission. Int. J. Dev. Neurosci. 80, 573–585. doi: 10.1002/jdn.10052, 32706909.

[ref2] Akyuz E. Arulsamy A. Aslan F. S. Sarisözen B. Guney B. Hekimoglu A. . (2025). An expanded narrative review of neurotransmitters on Alzheimer’s disease: the role of therapeutic interventions on neurotransmission. Mol. Neurobiol. 62, 1631–1674. doi: 10.1007/s12035-024-04333-y, 39012443. PMC11772559

[ref3] Alarcón T. A. Presti-Silva S. M. Simões A. P. T. Ribeiro F. M. Pires R. G. W. (2023). Molecular mechanisms underlying the neuroprotection of environmental enrichment in Parkinson’s disease. Neural Regen. Res. 18, 1450–1456. doi: 10.4103/1673-5374.360264, 36571341. PMC10075132

[ref4] Alcalá-Zúniga D. Espinoza-Torres E. Das R. K. Vargas M. Maldonado O. Benavides O. . (2024). Enriched environment contributes to the recovery from neurotoxin-induced Parkinson’s disease pathology. Mol. Neurobiol. 61, 6734–6753. doi: 10.1007/s12035-024-03951-w, 38349515. PMC11339186

[ref5] Andin J. Hallbeck M. Mohammed A. H. Marcusson J. (2007). Influence of environmental enrichment on steady-state mRNA levels for EAAC1, AMPA1 and NMDA2A receptor subunits in rat hippocampus. Brain Res. 1174, 18–27. doi: 10.1016/j.brainres.2007.06.101, 17854777.

[ref6] Arraes G. C. Barreto F. S. Vasconcelos G. S. de Carvalho Lima C. N. da Silva F. E. R. Ribeiro W. L. C. . (2024). Long-term environmental enrichment normalizes schizophrenia-like abnormalities and promotes hippocampal slc6a4 promoter demethylation in mice submitted to a two-hit model. Neuroscience 551, 205–216. doi: 10.1016/j.neuroscience.2024.05.023, 38843988.

[ref7] Balthazar J. Schöwe N. M. Cipolli G. C. Buck H. S. Viel T. A. (2018). Enriched environment significantly reduced senile plaques in a transgenic mice model of Alzheimer’s disease, improving memory. Front. Aging Neurosci. 10:288. doi: 10.3389/fnagi.2018.00288, 30319394. PMC6168651

[ref8] Baroncelli L. Braschi C. Spolidoro M. Begenisic T. Sale A. Maffei L. (2010). Nurturing brain plasticity: impact of environmental enrichment. Cell Death Differ. 17, 1092–1103. doi: 10.1038/cdd.2009.193, 20019745.

[ref9] Begega A. Jove C. I. López M. Moreno R.-D. (2023). Impact of environmental enrichment on the GABAergic neurons and glucocorticoid receptors in the hippocampus and nucleus accumbens of Wistar rats: pro-resilient effects. Brain Res. Bull. 200:110699. doi: 10.1016/j.brainresbull.2023.110699, 37406885.

[ref10] Begenisic T. Spolidoro M. Braschi C. Baroncelli L. Milanese M. Pietra G. . (2011). Environmental enrichment decreases GABAergic inhibition and improves cognitive abilities, synaptic plasticity, and visual functions in a mouse model of Down syndrome. Front. Cell. Neurosci. 5:29. doi: 10.3389/fncel.2011.00029, 22207837. PMC3245647

[ref11] Bolneo E. Chau P. Y. S. Noakes P. G. Bellingham M. C. (2022). Investigating the role of GABA in neural development and disease using mice lacking GAD67 or VGAT genes. Int. J. Mol. Sci. 23:7965. doi: 10.3390/ijms23147965 PMC9318753 35887307

[ref12] Bredy T. W. Zhang T. Y. Grant R. J. Diorio J. Meaney M. J. (2004). Peripubertal environmental enrichment reverses the effects of maternal care on hippocampal development and glutamate receptor subunit expression. Eur. J. Neurosci. 20, 1355–1362. doi: 10.1111/j.1460-9568.2004.03599.x, 15341607.

[ref13] Buschler A. Manahan-Vaughan D. (2017). Metabotropic glutamate receptor, mGlu5, mediates enhancements of hippocampal long-term potentiation after environmental enrichment in young and old mice. Neuropharmacology 115, 42–50. doi: 10.1016/j.neuropharm.2016.06.003, 27267685.

[ref14] Cabana-Domínguez J. Torrico B. Reif A. Fernàndez-Castillo N. Cormand B. (2022). Comprehensive exploration of the genetic contribution of the dopaminergic and serotonergic pathways to psychiatric disorders. Transl. Psychiatry 12:11. doi: 10.1038/s41398-021-01771-3 PMC8748838 35013130

[ref15] Caspi A. Hariri A. R. Holmes A. Uher R. Moffitt T. E. (2010). Genetic sensitivity to the environment: the case of the serotonin transporter gene and its implications for studying complex diseases and traits. Am. J. Psychiatry 167, 509–527. doi: 10.1176/appi.ajp.2010.09101452, 20231323. PMC2943341

[ref16] Charych E. I. Liu F. Moss S. J. Brandon N. J. (2009). GABAA receptors and their associated proteins: implications in the etiology and treatment of schizophrenia and related disorders. Neuropharmacology 57, 481–495. doi: 10.1016/j.neuropharm.2009.07.027, 19631671. PMC2836902

[ref17] Chattopadhyaya B. Di Cristo G. Wu C. Z. Knott G. Kuhlman S. Fu Y. . (2007). GAD67-mediated GABA synthesis and signaling regulate inhibitory synaptic innervation in the visual cortex. Neuron 54, 889–903. doi: 10.1016/j.neuron.2007.05.015, 17582330. PMC2077924

[ref18] Chen A. P. Chen L. Kim T. A. Xiong Q. (2021). Integrating the roles of midbrain dopamine circuits in behavior and neuropsychiatric disease. Biomedicines 9:647. doi: 10.3390/biomedicines9060647, 34200134. PMC8228225

[ref19] Chen Z.-R. Huang J.-B. Yang S.-L. Hong F.-F. (2022). Role of cholinergic signaling in Alzheimer’s disease. Molecules 27:1816. doi: 10.3390/molecules27061816 PMC8949236 35335180

[ref20] Chen G. Zhang Y. Li R. Jin L. Hao K. Rong J. . (2024). Environmental enrichment attenuates depressive-like behavior in maternal rats by inhibiting neuroinflammation and apoptosis and promoting neuroplasticity. Neurobiol. Stress 30:100624. doi: 10.1016/j.ynstr.2024.100624, 38524250. PMC10958482

[ref21] Cho H. Kang K. (2020). Effects of environmental enrichment on neurotrophins in an MPTP-induced Parkinson’s disease animal model: a randomized trial. Biol. Res. Nurs. 22, 506–513. doi: 10.1177/1099800420931183 32495646

[ref22] Chu B. Marwaha K. Sanvictores T. Awosika A. O. Ayers D. (2024). “Physiology, stress reaction,” in StatPearls (Treasure Island: StatPearls Publishing). 31082164

[ref23] Cipriani A. Furukawa T. A. Salanti G. Chaimani A. Atkinson L. Z. Ogawa Y. . (2018). Comparative efficacy and acceptability of 21 antidepressant drugs for the acute treatment of adults with major depressive disorder: a systematic review and network meta-analysis. Lancet 391, 1357–1366. doi: 10.1016/S0140-6736(17)32802-7, 29477251. PMC5889788

[ref24] Ciranna L. (2006). Serotonin as a modulator of glutamate-and GABA-mediated neurotransmission: implications in physiological functions and in pathology. Curr. Neuropharmacol. 4, 101–114. doi: 10.2174/157015906776359540, 18615128. PMC2430669

[ref25] Colavitta M. F. Grasso L. Barrantes F. J. (2023). Environmental enrichment in murine models and its translation to human factors improving conditions in Alzheimer disease. J. Prev. Alzheimer's Dis. 10, 287–300. doi: 10.14283/jpad.2023.5, 36946456.

[ref26] Costa G. A. de Gusmão Taveiros Silva N. K. Marianno P. Chivers P. Bailey A. Camarini R. (2023). Environmental enrichment increased bdnf transcripts in the prefrontal cortex: implications for an epigenetically controlled mechanism. Neuroscience 526, 277–289. doi: 10.1016/j.neuroscience.2023.07.001, 37419403.

[ref27] Coutens B. Lejards C. Bouisset G. Verret L. Rampon C. Guiard B. P. (2023). Enriched environmental exposure reduces the onset of action of the serotonin norepinephrin reuptake inhibitor venlafaxine through its effect on parvalbumin interneurons plasticity in mice. Transl. Psychiatry 13:227. doi: 10.1038/s41398-023-02519-x, 37365183. PMC10293286

[ref28] Cuellar-Santoyo A. O. Ruiz-Rodríguez V. M. Mares-Barbosa T. B. Patrón-Soberano A. Howe A. G. Portales-Pérez D. P. . (2023). Revealing the contribution of astrocytes to glutamatergic neuronal transmission. Front. Cell. Neurosci. 16:1037641. doi: 10.3389/fncel.2022.1037641, 36744061. PMC9893894

[ref29] da Silva R. P. B. Pinheiro I. L. da Silva R. K. B. Moretti E. C. de Oliveira Neto O. B. Ferraz-Pereira K. . (2024). Social isolation and post-weaning environmental enrichment effects on rat emotional behavior and serotonergic system. Int. J. Dev. Neurosci. 84, 265–280. doi: 10.1002/jdn.10324, 38526313.

[ref30] Darna M. Beckmann J. S. Gipson C. D. Bardo M. T. Dwoskin L. P. (2015). Effect of environmental enrichment on dopamine and serotonin transporters and glutamate neurotransmission in medial prefrontal and orbitofrontal cortex. Brain Res. 1599, 115–125. doi: 10.1016/j.brainres.2014.12.034, 25536304. PMC4344853

[ref31] de Sousa Fernandes M. S. Costa M. R. Badicu G. Yagin F. H. Santos G. C. J. da Costa J. M. . (2024). Can environmental enrichment modulate epigenetic processes in the central nervous system under adverse environmental conditions? A systematic review. Cell. Mol. Neurobiol. 44:69. doi: 10.1007/s10571-024-01506-0, 39432132. PMC11493835

[ref32] Del Arco A. Segovia G. Garrido P. de Blas M. Mora F. (2007). Stress, prefrontal cortex and environmental enrichment: studies on dopamine and acetylcholine release and working memory performance in rats. Behav. Brain Res. 176, 267–273. doi: 10.1016/j.bbr.2006.10.006 17097747

[ref33] Dong B. E. Chen H. Sakata K. (2020). BDNF deficiency and enriched environment treatment affect neurotransmitter gene expression differently across ages. J. Neurochem. 154, 41–55. doi: 10.1111/jnc.15017, 32222968. PMC7319906

[ref34] Echeverria V. Mendoza C. Iarkov A. (2023). Nicotinic acetylcholine receptors and learning and memory deficits in neuroinflammatory diseases. Front. Neurosci. 17:1179611. doi: 10.3389/fnins.2023.1179611, 37255751. PMC10225599

[ref35] Ellis J. R. Ellis K. A. Bartholomeusz C. F. Harrison B. J. Wesnes K. A. Erskine F. F. . (2006). Muscarinic and nicotinic receptors synergistically modulate working memory and attention in humans. Int. J. Neuropsychopharmacol. 9, 175–189. doi: 10.1017/S1461145705005407 15877932

[ref36] Fatemi S. H. Reutiman T. J. Folsom T. D. Thuras P. D. (2009). GABA A receptor downregulation in brains of subjects with autism. J. Autism Dev. Disord. 39, 223–230. doi: 10.1007/s10803-008-0646-7, 18821008. PMC2697059

[ref37] Ferreira-Vieira T. H. M Guimaraes I. R Silva F. M Ribeiro F. (2016). Alzheimer's disease: targeting the cholinergic system. Curr. Neuropharmacol. 14, 101–115. doi: 10.2174/1570159X13666150716165726, 26813123. PMC4787279

[ref38] Gabriel P. Mastracchio T.-A. Bordner K. Jeffrey R. (2020). Impact of enriched environment during adolescence on adult social behavior, hippocampal synaptic density and dopamine D2 receptor expression in rats. Physiol. Behav. 226:113133. doi: 10.1016/j.physbeh.2020.113133, 32795458.

[ref39] Gerhard D. M. Wohleb E. S. Duman R. S. (2016). Emerging treatment mechanisms for depression: focus on glutamate and synaptic plasticity. Drug Discov. Today 21, 454–464. doi: 10.1016/j.drudis.2016.01.016, 26854424. PMC4803609

[ref40] Goncalves L. V. Herlinger A. L. Ferreira T. A. A. Coitinho J. B. Pires R. G. W. Martins-Silva C. (2018). Environmental enrichment cognitive neuroprotection in an experimental model of cerebral ischemia: biochemical and molecular aspects. Behav. Brain Res. 348, 171–183. doi: 10.1016/j.bbr.2018.04.023, 29684474.

[ref41] Grilli M. Zappettini S. Zanardi A. Lagomarsino F. Pittaluga A. Zoli M. . (2009). Exposure to an enriched environment selectively increases the functional response of the pre-synaptic NMDA receptors which modulate noradrenaline release in mouse hippocampus. J. Neurochem. 110, 1598–1606. doi: 10.1111/j.1471-4159.2009.06265.x, 19723266.

[ref42] Grimm J. W. Glueck E. Ginder D. Hyde J. North K. Jiganti K. (2018). Sucrose abstinence and environmental enrichment effects on mesocorticolimbic DARPP32 in rats. Sci. Rep. 8:13174. doi: 10.1038/s41598-018-29625-x, 30181585. PMC6123458

[ref43] Gu B. (2025). New neurons, fewer seizures: environmental enrichment strengthens brain circuits. Epilepsy Curr. 25, 344–346. doi: 10.1177/15357597251352433, 40586043. PMC12198155

[ref44] Guiza-Zayas R. Uribe M. O. Palomera-Garfias N. Magaña-Saavedra E. Flores-Ramos M. (2025). Exploring the link between enriched environment and depression: insights from human participants. Actas Espanolas Psiquiatr. 53:979. doi: 10.62641/aep.v53i5.1927 PMC12538598 41117248

[ref45] Haas H. Panula P. (2003). The role of histamine and the tuberomamillary nucleus in the nervous system. Nat. Rev. Neurosci. 4, 121–130. doi: 10.1038/nrn1034 12563283

[ref46] Han P.-P. Han Y. Shen X.-Y. Gao Z.-K. Bi X. (2023). Enriched environment-induced neuroplasticity in ischemic stroke and its underlying mechanisms. Front. Cell. Neurosci. 17:1210361. doi: 10.3389/fncel.2023.1210361, 37484824. PMC10360187

[ref47] Harati H. Barbelivien A. Herbeaux K. Muller M.-A. Engeln M. Kelche C. . (2013). Lifelong environmental enrichment in rats: impact on emotional behavior, spatial memory vividness, and cholinergic neurons over the lifespan. AGE 35, 1027–1043. doi: 10.1007/s11357-012-9424-8, 22592932. PMC3705108

[ref48] Hasselmo M. E. (2006). The role of acetylcholine in learning and memory. Curr. Opin. Neurobiol. 16, 710–715. doi: 10.1016/j.conb.2006.09.002 PMC2659740 17011181

[ref49] He S. Ma J. Liu N. Yu X. (2010). Early enriched environment promotes neonatal GABAergic neurotransmission and accelerates synapse maturation. J. Neurosci. 30, 7910–7916. doi: 10.1523/JNEUROSCI.6375-09.2010, 20534839. PMC6632687

[ref50] Hensler J.G. (2010). “Serotonin in mood and emotion,” in Handbook of the Behavioral Neurobiology of Serotonin, Vol. 21, eds. C. P. Müller and B. L. Jacobs (Amsterdam: Elsevier), 367–378. doi: 10.1016/S1569-7339(10)70090-4

[ref51] Herselman M. Luo S. Lin L. Zhou X. Bobrovskaya L. (2026). Sex-dependent effects of environmental enrichment on mood-related behaviors, central and peripheral BDNF, and adrenal catecholamine enzymes in mice. Physiol. Rep. 14:e70870. doi: 10.14814/phy2.70870, 41981776. PMC13079431

[ref52] Hilario W. F. Herlinger A. L. Areal L. B. de Moraes L. S. Ferreira T. A. A. Andrade T. E. S. . (2016). Cholinergic and dopaminergic alterations in nigrostriatal neurons are involved in environmental enrichment motor protection in a mouse model of Parkinson’s disease. J. Mol. Neurosci. 60, 453–464. doi: 10.1007/s12031-016-0831-7, 27660217.

[ref53] Huang S. T. Ji N. N. Wang Y. Huang L. Zhou Z. M. Niu Y. W. . (2026). Environmental enrichment reverses ELS-induced visceral pain and depression through a prefrontal-limbic circuit involving CB1Rs. Commun. Biol. 9:871. doi: 10.1038/s42003-026-10105-2, 42032059. PMC13315944

[ref54] Hullinger R. O’Riordan K. Burger C. (2015). Environmental enrichment improves learning and memory and long-term potentiation in young adult rats through a mechanism requiring mGluR5 signaling and sustained activation of p70s6k. Neurobiol. Learn. Mem. 125, 126–134. doi: 10.1016/j.nlm.2015.08.006, 26341144. PMC4938427

[ref55] Inoue I. Yanai K. Kitamura D. Taniuchi I. Kobayashi T. Niimura K. . (1996). Impaired locomotor activity and exploratory behavior in mice lacking histamine H1 receptors. Proc. Natl. Acad. Sci. 93, 13316–13320. doi: 10.1073/pnas.93.23.13316 PMC24090 8917588

[ref56] Inzunza O. Bravo H. Fernández V. (2003). Environmental enrichment during early postnatal development decreases parvalbumin expression in the rat somatosensory cortex. Int. J. Morphol. 21, 181–189. doi: 10.4067/S0717-95022003000300001

[ref57] Jeyaraj S. E. Sivasangari K. García-Colunga J. Rajan K. E. (2021). Environmental enrichment enhances sociability by regulating glutamate signaling pathway through GR by epigenetic mechanisms in amygdala of Indian field mice Mus booduga. Gen. Comp. Endocrinol. 300:113641. doi: 10.1016/j.ygcen.2020.113641, 33017584.

[ref58] Jovanoski K. D. Duquenoy L. Mitchell J. Kapoor I. Treiber C. D. Croset V. . (2023). Dopaminergic systems create reward seeking despite adverse consequences. Nature 623, 356–365. doi: 10.1038/s41586-023-06671-8, 37880370. PMC10632144

[ref59] Kajita Y. Mushiake H. (2021). Heterogeneous GAD65 expression in subtypes of GABAergic neurons across layers of the cerebral cortex and hippocampus. Front. Behav. Neurosci. 15:750869. doi: 10.3389/fnbeh.2021.750869, 34803625. PMC8595203

[ref60] Kalia L. V. Lang A. E. (2015). Parkinson's disease. Lancet 386, 896–912. doi: 10.1016/S0140-6736(14)61393-3, 25904081.

[ref61] Karayol R. Medrihan L. Warner-Schmidt J. L. Fait B. W. Rao M. N. Holzner E. B. . (2021). Serotonin receptor 4 in the hippocampus modulates mood and anxiety. Mol. Psychiatry 26, 2334–2349. doi: 10.1038/s41380-020-00994-y, 33441982. PMC8275670

[ref62] Kempermann G. Gast D. Gage F. H. (2002). Neuroplasticity in old age: sustained fivefold induction of hippocampal neurogenesis by long-term environmental enrichment. Ann. Neurol. 52, 135–143. doi: 10.1002/ana.10262, 12210782.

[ref63] Khalil M. H. (2024). Environmental enrichment: a systematic review on the effect of a changing spatial complexity on hippocampal neurogenesis and plasticity in rodents, with considerations for translation to urban and built environments for humans. Front. Neurosci. 18:1368411. doi: 10.3389/fnins.2024.1368411, 38919908. PMC11196820

[ref64] Kilb W. Kirischuk S. (2022). GABA release from astrocytes in health and disease. Int. J. Mol. Sci. 23:15859. doi: 10.3390/ijms232415859, 36555501. PMC9784789

[ref65] Kim M.-S. Yu J. H. Kim C. H. Choi J. Y. Seo J. H. Lee M.-Y. . (2016). Environmental enrichment enhances synaptic plasticity by internalization of striatal dopamine transporters. J. Cereb. Blood Flow. Metab. 36, 2122–2133. doi: 10.1177/0271678X15613525, 26661218. PMC5363660

[ref66] Koh S. Magid R. Chung H. Stine C. D. Wilson D. N. (2007). Depressive behavior and selective downregulation of serotonin receptor expression after early-life seizures: reversal by environmental enrichment. Epilepsy Behav. 10, 26–31. doi: 10.1016/j.yebeh.2006.11.008, 17196883. PMC1876703

[ref67] Kubota T. (2016). Epigenetic alterations induced by environmental stress associated with metabolic and neurodevelopmental disorders. Environ. Epigenetics 2:dvw017. doi: 10.1093/eep/dvw017, 29492297. PMC5804531

[ref68] Kudryavtseva N. Smagin D. Kovalenko I. Galyamina A. Vishnivetskaya G. Babenko V. . (2017). Serotonergic genes in the development of anxiety/depression-like state and pathology of aggressive behavior in male mice: RNA-seq data. Mol. Biol. 51, 251–262. doi: 10.7868/S0026898417020136 28537235

[ref69] Lee E. H. Hsu W. Ma Y. Lee P. Chao C. (2003). Enrichment enhances the expression of sgk, a glucocorticoid-induced gene, and facilitates spatial learning through glutamate AMPA receptor mediation. Eur. J. Neurosci. 18, 2842–2852. doi: 10.1111/j.1460-9568.2003.03032.x 14656333

[ref70] Leger M. Paizanis E. Dzahini K. Quiedeville A. Bouet V. Cassel J.-C. . (2015). Environmental enrichment duration differentially affects behavior and neuroplasticity in adult mice. Cereb. Cortex 25, 4048–4061. doi: 10.1093/cercor/bhu119, 24904072.

[ref71] Levinson A. Young T. (2022). “Neurobiology of bipolar disorder,” in Neuroscience in the 21st Century: From Basic to Clinical, 3rd Edn, eds. D. W. Pfaff, N. D. Volkow, and J. L. R. Rubenstein (Cham: Springer Nature Switzerland AG), 4417–4436. doi: 10.10007/978-1-4614-6434-1_117-3

[ref72] Li Z. Chen L. Fei F. Wang W. Yang L. Wang Y. . (2024). Enriched environment reduces seizure susceptibility via entorhinal cortex circuit augmented adult neurogenesis. Adv. Sci. 11:2410927. doi: 10.1002/advs.202410927 PMC11633471 39435757

[ref73] Li C. Niu W. Jiang C. H. Hu Y. (2007). Effects of enriched environment on gene expression and signal pathways in cortex of hippocampal CA1 specific NMDAR1 knockout mice. Brain Res. Bull. 71, 568–577. doi: 10.1016/j.brainresbull.2006.11.011, 17292799.

[ref74] Liberati A. Altman D. G. Tetzlaff J. Mulrow C. Gøtzsche P. C. Ioannidis J. P. . (2009). The PRISMA statement for reporting systematic reviews and meta-analyses of studies that evaluate health care interventions: explanation and elaboration. PLoS Med. 6:e1000100. doi: 10.1371/journal.pmed.1000100, 19621070. PMC2707010

[ref75] Lin Y. Yao M. Wu H. Wu F. Cao S. Ni H. . (2021). Environmental enrichment implies GAT-1 as a potential therapeutic target for stroke recovery. Theranostics 11:3760. doi: 10.7150/thno.53316, 33664860. PMC7914370

[ref76] Martínez-Torres N. I. Cárdenas-Bedoya J. Vázquez-Torres B. M. Torres-Mendoza B. M. (2024). Environmental enrichment and cerebrolysin improve motor and cognitive performance in a rat model of stroke, in conjunction with an increase in hippocampal AMPA but not NMDA receptor subunits. Brain Res. 1825:148694. doi: 10.1016/j.brainres.2023.148694, 38048977.

[ref77] Maurer S. V. Williams C. L. (2017). The cholinergic system modulates memory and hippocampal plasticity via its interactions with non-neuronal cells. Front. Immunol. 8:1489. doi: 10.3389/fimmu.2017.01489, 29167670. PMC5682336

[ref78] McAlees J. W. Smith L. T. Erbe R. S. Jarjoura D. Ponzio N. M. Sanders V. M. (2011). Epigenetic regulation of beta2-adrenergic receptor expression in TH1 and TH2 cells. Brain Behav. Immun. 25, 408–415. doi: 10.1016/j.bbi.2010.10.019, 21047549. PMC3073579

[ref79] McGrath T. Baskerville R. Rogero M. Castell L. (2022). Emerging evidence for the widespread role of glutamatergic dysfunction in neuropsychiatric diseases. Nutrients 14:917. doi: 10.3390/nu14050917, 35267893. PMC8912368

[ref80] Mei Y. Zhang X.-X. Xiao-Cui F. Xia B. (2020). Enriched environment alleviates post-stroke cognitive impairment through enhancing α7-nAChR expression in rats. Arq. Neuro-Psiquiatria 78, 603–610. doi: 10.1590/0004-282X20200081, 33111849.

[ref81] Meldrum B. S. (2000). Glutamate as a neurotransmitter in the brain: review of physiology and pathology. J. Nutr. 130, 1007S–1015S. doi: 10.1093/jn/130.4.1007S 10736372

[ref82] Mickley G. A. (1986). Histamine H_2_ receptors mediate morphine-induced locomotor hyperactivity of the C57BL/6J mouse. Behav. Neurosci. 100:79. doi: 10.1037//0735-7044.100.1.79, 3954883.

[ref83] Mlynarik M. Johansson B. B. Jezova D. (2004). Enriched environment influences adrenocortical response to immune challenge and glutamate receptor gene expression in rat hippocampus. Ann. N. Y. Acad. Sci. 1018, 273–280. doi: 10.1196/annals.1296.032, 15240378.

[ref84] Moher D. Liberati A. Tetzlaff J. Altman D. G. PRISMA Group (2009). Preferred reporting items for systematic reviews and meta-analyses: the PRISMA statement. PLoS Med. 6:e1000097. doi: 10.1371/journal.pmed.1000097, 21603045. PMC3090117

[ref85] Mohsen A. Yoshikawa T. Miura Y. Nakamura T. Naganuma F. Shibuya K. . (2014). Mechanism of the histamine H3 receptor-mediated increase in exploratory locomotor activity and anxiety-like behaviours in mice. Neuropharmacology 81, 188–194. doi: 10.1016/j.neuropharm.2014.02.003, 24530460.

[ref86] Nakamura Y. Ishimaru K. Shibata S. Nakao A. (2017). Regulation of plasma histamine levels by the mast cell clock and its modulation by stress. Sci. Rep. 7:39934. doi: 10.1038/srep39934 PMC5225447 28074918

[ref87] Nam Y. Kim S. Park Y. H. Kim B. H. Shin S. J. Leem S. H. . (2024). Investigating the impact of environmental enrichment on proteome and neurotransmitter-related profiles in an animal model of Alzheimer's disease. Aging Cell 23:e14231. doi: 10.1111/acel.14231, 38952076. PMC11488327

[ref88] Neves R. C. Figueiredo R. C. Faria-Melibeu A. C. (2024). The potential role of enrichment environment on plasticity in Alzheimer's disease models: insights about therapeutic approaches. Curr. Alzheimer Res. 21, 457–469. doi: 10.2174/0115672050348227241128095209, 39623716.

[ref89] Newman-Tancredi A. Albert P. R. Sumiyoshi T.. (2012). Gene polymorphism at serotonin 5-HT1A receptors: moving towards personalized medicine for psychosis and mood deficits. Schizophr. Res.: Recent Adv., 337–358.

[ref90] Nichols J. Jakkamsetti V. Salgado H. Dinh L. Kilgard M. Atzori M. (2007). Environmental enrichment selectively increases glutamatergic responses in layer II/III of the auditory cortex of the rat. Neuroscience 145, 832–840. doi: 10.1016/j.neuroscience.2006.12.061 PMC2824591 17291690

[ref91] Nieoullon A. (2002). Dopamine and the regulation of cognition and attention. Prog. Neurobiol. 67, 53–83. doi: 10.1016/s0301-0082(02)00011-4 12126656

[ref92] Paban V. Chambon C. Manrique C. Touzet C. Alescio-Lautier B. (2011). Neurotrophic signaling molecules associated with cholinergic damage in young and aged rats: environmental enrichment as potential therapeutic agent. Neurobiol. Aging 32, 470–485. doi: 10.1016/j.neurobiolaging.2009.03.010, 19398249.

[ref93] Pajarillo E. Digman A. Nyarko-Danquah I. Son D.-S. Soliman K. F. Aschner M. . (2021). Astrocytic transcription factor REST upregulates glutamate transporter EAAT2, protecting dopaminergic neurons from manganese-induced excitotoxicity. J. Biol. Chem. 297:101372. doi: 10.1016/j.jbc.2021.101372, 34756885. PMC8626589

[ref94] Peitl V. Ivančić Ravlić I. Golubić Zatezalo V. (2020). Genetic polymorphisms of the dopamine and serotonin systems in schizophrenia in relation to violence and aggression. Arch. Psychiatry Res.: Int. J. Psychiatry Relat. Sci. 56, 63–74. doi: 10.20471/may.2020.56.01.06

[ref95] Peixoto L. Abel T. (2013). The role of histone acetylation in memory formation and cognitive impairments. Neuropsychopharmacology 38, 62–76. doi: 10.1038/npp.2012.86 PMC3521994 22669172

[ref96] Penney J. Tsai L.-H. (2014). Histone deacetylases in memory and cognition. Sci. Signal. 7, re12. doi: 10.1126/scisignal.aaa0069 25492968

[ref97] Pintori N. Piva A. Mottarlini F. Díaz F. C. Maggi C. Caffino L. . (2024). Brief exposure to enriched environment rapidly shapes the glutamate synapses in the rat brain: a metaplastic fingerprint. Eur. J. Neurosci. 59, 982–995. doi: 10.1111/ejn.16279, 38378276.

[ref98] Pourhamzeh M. Moravej F. G. Arabi M. Shahriari E. Mehrabi S. Ward R. . (2022). The roles of serotonin in neuropsychiatric disorders. Cell. Mol. Neurobiol. 42, 1671–1692. doi: 10.1007/s10571-021-01064-9, 33651238. PMC11421740

[ref99] Puglisi-Allegra S. Andolina D. (2015). Serotonin and stress coping. Behav. Brain Res. 277, 58–67. doi: 10.1016/j.bbr.2014.07.052 25108244

[ref100] Ragu Varman D. Marimuthu G. Emmanuvel Rajan K. (2013). Environmental enrichment upregulates micro-RNA-183 and alters acetylcholinesterase splice variants to reduce anxiety-like behavior in the little Indian field mouse (Mus booduga) J. Neurosci. Res. 91, 426–435. doi: 10.1002/jnr.23165, 23184316.

[ref101] Ren Z.-Y. Han B.-Y. Zhao L.-Y. Lou X.-J. Tao Y.-X. Zhang G.-F. . (2025). Environmental enrichment alleviates neuropathic pain-associated anxiety by enhancing the function of parvalbumin interneurons in the anterior cingulate cortex. Sci. Rep. 15:10131. doi: 10.1038/s41598-025-95220-6, 40128579. PMC11933386

[ref102] Renoir T. Pang T. Y. Mo C. Chan G. Chevarin C. Lanfumey L. . (2013). Differential effects of early environmental enrichment on emotionality related behaviours in Huntington's disease transgenic mice. J. Physiol. 591, 41–55. doi: 10.1113/jphysiol.2012.239798, 23045340. PMC3630770

[ref103] Rogers J. Li S. Lanfumey L. Hannan A. J. Renoir T. (2017). Environmental enrichment reduces innate anxiety with no effect on depression-like behaviour in mice lacking the serotonin transporter. Behav. Brain Res. 332, 355–361. doi: 10.1016/j.bbr.2017.06.009, 28629963.

[ref104] Rogers J. Renoir T. Hannan A. J. (2019). Gene-environment interactions informing therapeutic approaches to cognitive and affective disorders. Neuropharmacology 145, 37–48. doi: 10.1016/j.neuropharm.2017.12.038, 29277490.

[ref105] Rogers J. Vo U. Buret L. Pang T. Meiklejohn H. Zeleznikow-Johnston A. . (2016). Dissociating the therapeutic effects of environmental enrichment and exercise in a mouse model of anxiety with cognitive impairment. Transl. Psychiatry 6:e794. doi: 10.1038/tp.2016.52, 27115125. PMC4872410

[ref106] Ross J. A. Van Bockstaele E. J. (2021). The locus coeruleus-norepinephrine system in stress and arousal: unraveling historical, current, and future perspectives. Front. Psychiatry 11:601519. doi: 10.3389/fpsyt.2020.601519, 33584368. PMC7873441

[ref107] Ryding S. Garnham L. C. Abbey-Lee R. N. Petkova I. Kreshchenko A. Løvlie H. (2021). Impulsivity is affected by cognitive enrichment and links to brain gene expression in red junglefowl chicks. Anim. Behav. 178, 195–207. doi: 10.1016/j.anbehav.2021.06.007

[ref108] Saadati H. Ghaheri S. Sadegzadeh F. Sakhaie N. Abdollahzadeh M. (2023). Beneficial effects of enriched environment on behavior, cognitive functions, and hippocampal brain-derived neurotrophic factor level following postnatal serotonin depletion in male rats. Int. J. Dev. Neurosci. 83, 67–79. doi: 10.1002/jdn.10238, 36342785.

[ref109] Sahini S. N. M. Hazalin N. A. M. N. Srikumar B. N. Chellammal H. S. J. Singh G. K. S. (2024). Environmental enrichment improves cognitive function, learning, memory and anxiety-related behaviours in rodent models of dementia: implications for future study. Neurobiol. Learn. Mem. 208:107880. doi: 10.1016/j.nlm.2023.107880, 38103676.

[ref110] Salinas-Velarde I. D. Bernal-Morales B. Pacheco-Cabrera P. Sánchez-Aparicio P. Pascual-Mathey L. I. Venebra-Muñoz A. (2021). Lower ΔFosB expression in the dopaminergic system after stevia consumption in rats housed under environmental enrichment conditions. Brain Res. Bull. 177, 172–180. doi: 10.1016/j.brainresbull.2021.10.001, 34624462.

[ref111] Sbrini G. Brivio P. Bosch K. Homberg J. R. Calabrese F. (2020). Enrichment environment positively influences depression-and anxiety-like behavior in serotonin transporter knockout rats through the modulation of neuroplasticity, spine, and GABAergic markers. Genes 11:1248. doi: 10.3390/genes11111248, 33114023. PMC7690660

[ref112] Schlett K. (2006). Glutamate as a modulator of embryonic and adult neurogenesis. Curr. Top. Med. Chem. 6, 949–960. doi: 10.2174/156802606777323665, 16787269.

[ref113] Segovia G. Yagüe A. G. García-Verdugo J. M. Mora F. (2006). Environmental enrichment promotes neurogenesis and changes the extracellular concentrations of glutamate and GABA in the hippocampus of aged rats. Brain Res. Bull. 70, 8–14. doi: 10.1016/j.brainresbull.2005.11.005, 16750477.

[ref114] Šimić G. Leko M. B. Wray S. Harrington C. R. Delalle I. Jovanov-Milošević N. . (2017). Monoaminergic neuropathology in Alzheimer’s disease. Prog. Neurobiol. 151, 101–138. doi: 10.1016/j.pneurobio.2016.04.001, 27084356. PMC5061605

[ref115] Singhal G. Baune B. T. (2017). Microglia: an interface between the loss of neuroplasticity and depression. Front. Cell. Neurosci. 11:270. doi: 10.3389/fncel.2017.00270, 28943841. PMC5596091

[ref116] Singhal G. Jaehne E. J. Corrigan F. Baune B. T. (2014). Cellular and molecular mechanisms of immunomodulation in the brain through environmental enrichment. Front. Cell. Neurosci. 8:97. doi: 10.3389/fncel.2014.00097, 24772064. PMC3982075

[ref117] Soga T. Teo C. H. Parhar I. (2021). Genetic and epigenetic consequence of early-life social stress on depression: role of serotonin-associated genes. Front. Genet. 11:601868. doi: 10.3389/fgene.2020.601868, 33584798. PMC7874148

[ref118] Sowndharya S. Rajan K. E. (2026). Interaction of serotonin transporter and TGFB1 facilitates histone serotonylation-mediated synaptic plasticity following environmental enrichment in stressed mouse Mus booduga. Epigenet. Chromatin 19:9. doi: 10.1186/s13072-025-00653-y, 41486372. PMC12865968

[ref119] Takamura N. Nakagawa S. Masuda T. Boku S. Kato A. Song N. . (2014). The effect of dopamine on adult hippocampal neurogenesis. Prog. Neuro-Psychopharmacol. Biol. Psychiatry 50, 116–124. doi: 10.1016/j.pnpbp.2013.12.011 24374069

[ref120] Tashiro M. Mochizuki H. Iwabuchi K. Sakurada Y. Itoh M. Watanabe T. . (2002). Roles of histamine in regulation of arousal and cognition: functional neuroimaging of histamine H1 receptors in human brain. Life Sci. 72, 409–414. doi: 10.1016/s0024-3205(02)02276-2, 12467881.

[ref121] Terrier C. Greco-Vuilloud J. Cavelius M. Thevenet M. Mandairon N. Didier A. . (2024). Long-term olfactory enrichment promotes non-olfactory cognition, noradrenergic plasticity and remodeling of brain functional connectivity in older mice. Neurobiol. Aging 136, 133–156. doi: 10.1016/j.neurobiolaging.2024.01.011, 38364691.

[ref122] Thanos P. K. Hamilton J. O'Rourke J. R. Napoli A. Febo M. Volkow N. D. . (2016). Dopamine D2 gene expression interacts with environmental enrichment to impact lifespan and behavior. Oncotarget 7:19111. doi: 10.18632/oncotarget.8088, 26992232. PMC4991369

[ref123] Torres R. F. Llontop N. Espinoza C. S. Kerr B. (2025). “Environmental enrichment and epigenetic changes in the brain: from the outside to the deep inside,” in Neuroepigenetics Mechanisms in Health and Disease, Vol. 108, eds. D. W. Pfaff, N. D. Volkow, and J. L. R. Rubenstein (Cham: Springer Nature Switzerland AG), 4417–4436. doi: 10.1007/978-1-4614-6434-1_117-3 39820864

[ref124] Traynelis S. F. Wollmuth L. P. McBain C. J. Menniti F. S. Vance K. M. Ogden K. K. . (2010). Glutamate receptor ion channels: structure, regulation, and function. Pharmacol. Rev. 62, 405–496. doi: 10.1124/pr.109.002451, 20716669. PMC2964903

[ref125] Vaidya V. Vadodaria K. Jha S. (2007). Neurotransmitter regulation of adult neurogenesis: putative therapeutic targets. CNS Neurol. Disord. Targets (Former. Curr. Drug. Targets-CNS Neurol. Disord.) 6, 358–374. doi: 10.2174/187152707783220910 18045164

[ref126] Vargas-Romero F. González-Barrios R. Guerra-Calderas L. Escobedo-Avila I. Cortés-Pérez D. López-Ornelas A. . (2019). Histamine modulates midbrain dopamine neuron differentiation through the regulation of epigenetic marks. Front. Cell. Neurosci. 13:215. doi: 10.3389/fncel.2019.00215, 31178697. PMC6536891

[ref127] Vashisht A. Adamson G. Gacso Z. Slama J. Freund M. Vinod S. . (2025). Environmental enrichment attenuates reinstatement of heroin seeking and reverses heroin-induced upregulation of mesolimbic ghrelin receptors. Drug Alcohol Depend. 270:112635. doi: 10.1016/j.drugalcdep.2025.112635, 40022817. PMC11908936

[ref128] Vega-Rosas A. Flores-Ramos M. Ramírez-Rodríguez G. B. (2024). Association between the enriched environment level and serum brain-derived neurotrophic factor (BDNF) in patients with major depressive disorder. Brain Sci. 14:1137. doi: 10.3390/brainsci14111137, 39595900. PMC11592353

[ref129] Venditti S. Verdone L. Reale A. Vetriani V. Caserta M. Zampieri M. (2020). Molecules of silence: effects of meditation on gene expression and epigenetics. Front. Psychol. 11:1767. doi: 10.3389/fpsyg.2020.01767, 32849047. PMC7431950

[ref130] Wang H. Li J.-T. Liu D.-N. Zhang X.-Q. Sun M. Zhang C.-C. . (2025). Environmental enrichment improves deficits in hippocampal neuroplasticity and cognition in prenatally aripiprazole-exposed mouse offspring. Transl. Psychiatry 15:102. doi: 10.1038/s41398-025-03335-1, 40148276. PMC11950651

[ref131] Wassouf Z. Hentrich T. Samer S. Rotermund C. Kahle P. J. Ehrlich I. . (2018). Environmental enrichment prevents transcriptional disturbances induced by alpha-synuclein overexpression. Front. Cell. Neurosci. 12:112. doi: 10.3389/fncel.2018.00112, 29755323. PMC5932345

[ref132] Xie J. Luo Y. Wei L. Fan H. Wang Y. Wang Q. . (2024). Effects of environmental enrichment on GLUT expression in the visual cortex of amblyopic rats. Brain Res. 1836:148933. doi: 10.1016/j.brainres.2024.148933, 38604554.

[ref133] Xu Y.-Y. Du J.-J. Du Z.-A. Liu D.-L. Chen K.-D. Che Y.-C. . (2025). Enriched environment prevented glutamate receptor loss in the prefrontal cortex to alleviate CUMS-induced depression-like behaviors. J. Affect. Disord. 395:120634. doi: 10.1016/j.jad.2025.120634, 41237964.

[ref134] Xu H. Li B. Li L. Fan Z. Gong X. Wu L. . (2022). Environmental enrichment mitigates PTSD-like behaviors in adult male rats exposed to early life stress by regulating histone acetylation in the hippocampus and amygdala. J. Psychiatr. Res. 155, 120–136. doi: 10.1016/j.jpsychires.2022.07.067 36029624

[ref135] Xu H. Rajsombath M. M. Weikop P. Selkoe D. J. (2018). Enriched environment enhances β-adrenergic signaling to prevent microglia inflammation by amyloid-β. EMBO Mol. Med. 10: e8931. doi: 10.15252/emmm.201808931, 30093491. PMC6127891

[ref136] Yeung R. K. Xiang Z.-H. Tsang S.-Y. Li R. Ho T. Y. Li Q. . (2018). Gabrb2-knockout mice displayed schizophrenia-like and comorbid phenotypes with interneuron–astrocyte–microglia dysregulation. Transl. Psychiatry 8:128. doi: 10.1038/s41398-018-0176-9, 30013074. PMC6048160

[ref137] Yuan Z.-Y. Yang J. Ma X.-W. Wang Y.-Y. Wang M.-W. (2018). Enriched environment elevates expression of growth associated protein-43 in the substantia nigra of SAMP8 mice. Neural Regen. Res. 13, 1988–1994. doi: 10.4103/1673-5374.239447, 30233074. PMC6183044

[ref138] Zajac M. S. Renoir T. Perreau V. M. Li S. Adams W. van den Buuse M. . (2018). Short-term environmental stimulation spatiotemporally modulates specific serotonin receptor gene expression and behavioral pharmacology in a sexually dimorphic manner in Huntington's disease transgenic mice. Front. Mol. Neurosci. 11:433. doi: 10.3389/fnmol.2018.00433, 30618600. PMC6295568

[ref139] Zhang Y. Crofton E. J. Li D. Lobo M. K. Fan X. Nestler E. J. . (2014). Overexpression of DeltaFosB in nucleus accumbens mimics the protective addiction phenotype, but not the protective depression phenotype of environmental enrichment. Front. Behav. Neurosci. 8:297. doi: 10.3389/fnbeh.2014.00297, 25221490. PMC4148937

[ref140] Zhang W. Jiang G. Kang H. Wang J. Liu Z. Wang Z. . (2024). Environmental enrichment exposure alleviates geriatric depressive-like symptoms through regulating neurogenesis and neuroinflammation. Environ. Health 3, 259–270. doi: 10.1021/envhealth.4c00186, 40144319. PMC11934201

[ref141] Zhang Y. Liu W. Huang T. Liu L. Chen X. (2025). Enriched environment inhibits GLT-1 ubiquitination by downregulating SMURF1 to attenuate ischemic brain injury induced excitotoxicity. Cell Biosci. 15:124. doi: 10.1186/s13578-025-01457-z, 40877964. PMC12395883

[ref142] Zhao H. Mao X. Zhu C. Zou X. Peng F. Yang W. . (2022). GABAergic system dysfunction in autism spectrum disorders. Front. Cell Dev. Biol. 9:781327. doi: 10.3389/fcell.2021.781327 PMC8858939 35198562

[ref143] Zhu X. Grace A. A. (2022). Use of prepubertal environment enrichment to prevent dopamine dysregulation in a neurodevelopmental rat model of schizophrenia risk. STAR Protoc. 3:101215. doi: 10.1016/j.xpro.2022.101215, 35265862. PMC8899041

